# Global, regional, and national burden of headache disorders, 1990–2021, and projections to 2050: a comprehensive analysis of the global burden of disease study 2021

**DOI:** 10.3389/fneur.2025.1674946

**Published:** 2025-10-29

**Authors:** Linxue Shen, Hui Li, Kuihua Wang, Ting Guo, Jianxin Ye

**Affiliations:** ^1^Department of Neurology, Fuzong Clinical Medical College of Fujian Medical University, Fuzhou, China; ^2^Department of Radiation Oncology, Clinical Oncology School of Fujian Medical University, Fujian Cancer Hospital, Fuzhou, China; ^3^Department of Neurology, 900th Hospital of PLA Joint Logistic Support Force, Fuzhou, China; ^4^Department of Infectious Disease, Fuzong Clinical Medical College of Fujian Medical University, Fuzhou, China

**Keywords:** headache disorders, migraines, tension-type headaches, global burden, trend and prediction, health inequalities

## Abstract

**Introduction:**

Headache disorders, including migraines and tension-type headaches, are prevalent and debilitating conditions that affect millions of people worldwide. And this analysis aims to inform evidence-based interventions and policies for alleviating the burden of headache disorders on individuals and societies.

**Methods:**

We analyzed headache data from the Global Burden of Disease Study 2021 report, examining the prevalence, incidence, and disability-adjusted life years across 204 countries and territories over 32 years. The data were stratified according to age, sex, year, geographical region, and socio-demographic Index (SDI). We employed the estimated average percentage change calculation to assess the temporal trends of these indicators. A detailed analysis of health inequities was conducted using decomposition analysis, the pyramid model, slope index, and concentration index. The Bayesian Age-Period-Cohort model was used to forecast the disease burden over the next 26 years.

**Results:**

By 2021, the global incidence of headache disorders was estimated to be 809.2 million, with tension-type headaches being the most prevalent. Age-standardized incidence rates, prevalence rates, and DALYs have exhibited an overall upward trend since 1990. The health inequality analysis revealed a diminishing disparity in the burden of headache disorders across countries with varying SDI scores. Predictions indicate a continuous increase in headache disorders, potentially reaching over 3.5 billion by 2050 based on current trends.

**Conclusion:**

The burden of headache disorders is increasing, with important implications for global health and the economy. The rising predictions highlight the potential need for targeted public health strategies and interventions.

## 1 Introduction

Headache disorders, including migraine and tension-type headaches, are among the most common and disabling neurological conditions worldwide. The International Classification of Diseases defines headaches as painful conditions affecting upper region of the head that can severely affect daily functioning and quality of life (1). In the Global Burden of Disease Study (GBD) 2021, headache disorders are ranked as the 15th leading cause of disease burden, measured in disability-adjusted life years (DALYs) across all age groups. Among young adults, migraine was the second largest contributor to DALYs (2). Age-standardized DALYs caused by migraines rank third among neurological diseases (3). Globally, the burden of migraine has notably increased among women of childbearing age, particularly in the 45–49 age group (4). Between 1990 and 2021, migraine prevalence rose substantially in the global population aged 15–39. By 2021, an estimated 593.8 million cases were reported, a 39.52% increase from 425.6 million in 1990 (5). Nevertheless, these studies have not analyzed headache disorders, including tension-type headaches.

Headache disorders can severely impact individuals’ lives, leading to increased absenteeism from work and school as well as substantial economic losses from reduced productivity and healthcare costs (6, 7). The onset and progression of headache disorders can be attributed to a biopsychosocial model, encompassing biological (e.g., obesity, sleep abnormalities), psychological (e.g., stress, psychiatric disorders), and social determinants (8–11). A considerable proportion of these factors are amenable to intervention, which can alleviate the occurrence of headaches. Therefore, understanding the burden and trends in headache disorders is essential for public health planning and research, healthcare delivery, and research priorities. This study aimed to systematically analyze the global, regional, and national burden of headache disorders, including migraines and tension-type headaches, from 1990 to 2021 and predict future estimates for 2050 using data from the GBD 2021. These analyses provide a crucial evidence base to inform long-term global health policy and strategic health system planning, while also offering essential large-scale population context that guides the clinical work and research priorities of neurologists, headache specialists, and neuroscientists. By examining the incidence, prevalence, and DALYs of these disorders, this study seeks to contribute to evidence-based interventions and policies for alleviating the burden of headache disorders on individuals and societies.

## 2 Methods

### 2.1 Data source

This study employed data from the 2021 GBD, Injuries, and Risk Factors Study, which offers comprehensive, standardized information on disease burden, risk factors, and mortality derived from health surveys, vital registration systems, and published literature across 7 GBD super regions and 204 countries. A critical methodological limitation of the GBD estimates is the heavy reliance on modeled data to fill gaps in countries with sparse or non-existent empirical data, particularly in low-SDI regions (2).

### 2.2 Study design

We adopted a cross-sectional approach to analyze the burden, decomposition, and associated health inequalities of headache disorders and predicted the disease burden of headache disorders by 2050. The measured outcomes included incidence, prevalence, age-standardized rates (ASR), and DALYs.

### 2.3 Statistical analyses

#### 2.3.1 Global burden and trends

Joinpoint regression analysis is a statistical technique to identify turning points in time-series data trends. Compared to simple linear regression which assumes a constant trend over the entire period, joinpoint regression allows for the detection of multiple segments with statistically distinct slopes, thereby providing a more nuanced understanding of temporal patterns. It enables researchers to ascertain whether a particular health indicator is experiencing a continuous rise, decline, or stability over a certain period and the precise timing of these changes. We applied the joinpoint regression model by selecting five turning points to divide the data into six segments to analyze temporal trends in global incidence, prevalence, and DALYs for headache disorders and their two subclasses.

#### 2.3.2 Regional and national burden and trends

Estimated average percentage change (EAPC) is a statistical measure in epidemiology that quantifies the average annual percentage change in health indicators or disease rates over a specific period. It is commonly used to assess temporal trends and compare the magnitude of changes across populations or time intervals. We compiled the numbers and ASR (per 100,000 individuals) of incidence, prevalence, and DALYs at the global, age-group, sociodemographic index (SDI), and GBD super-region levels for 2021. Trend changes from 1990 to 2021, EAPC for the ASR of incident cases, DALYs, and prevalence were calculated to understand the rate of change over the study period. We used world maps to present the rates and numbers of incidence, prevalence, DALYs, and EAPC for 204 countries and regions.

#### 2.3.3 Sex and age burden

The Pyramid Model is a graphical tool for showcasing and interpreting the distribution and hierarchical structure of data, enabling clear visualization of patterns, trends, and proportional relationships. We used this model to present the sex distribution of headache occurrence across different age groups. The horizontal axis usually denotes sex (male and female), the vertical axis signifies the number of headaches across various age ranges, and the width of the pyramid symbolizes the number of headaches.

#### 2.3.4 Health inequality

Decomposition analysis was performed to elucidate the roles of population size, aging, and epidemiological shifts in shaping disease burden across varying SDI levels, aiming to identify the key determinants influencing disease burden at different stages of development.

The absolute health inequality index slope measures health inequality by assessing the slope of a line that plots health outcomes against the SDI. A steeper slope signifies greater health disparities with worse outcomes in the lower SDI groups.

In health equity analysis, the intercept refers to the health outcome value when the SDI is at its lowest level. It represents the baseline health status of the most disadvantaged socioeconomic group and shows the expected health level if all other factors are constant.

The Relative Health Inequity Concentration Index assesses the concentration of health outcomes across different socioeconomic strata, examining the distribution of health outcomes relative to the SDI distribution. A high concentration index suggests that certain health outcomes are more prevalent in specific socioeconomic groups, indicating potential health inequalities not solely attributable to the overall population’s health level.

#### 2.3.5 Prediction

The Age-Period-Cohort (APC) model is a statistical framework for analyzing age, period, and cohort effects within time-series data. In this model, the age effect refers to the biological or physiological changes that occur with aging. The period effect pertains to external factors that change over time, including socioeconomic conditions, advancements in medical technology, and epidemics. The cohort effect reflects the unique characteristics of individuals born in the same year and may be influenced by historical, cultural, and socioeconomic factors. Bayesian Age-Period-Cohort (BAPC) models are constructed within a Bayesian statistical framework, enhancing the APC model by providing more robust estimates, addressing the issue of collinearity, and quantifying the uncertainty of the model parameters. This enhancement makes the BAPC models more effective and useful for analyzing time-series health data. We used BAPC to forecast the number and rate of the incidence, prevalence, and DALYs associated with headache disorders by the year 2050.

### 2.4 Statistical software

Stata/MP 17 (64-bit) calculated concentration indices with the “conindex” package. R 4.4.1 performed other statistical analyses and graphics using R packages like “dplyr,” “ggplot2,” and “MASS” for data manipulation, visualization, and regression. Statistical significance was defined as *p*-values < 0.05.

## 3 Result

### 3.1 Global burden

**Figure 1:**
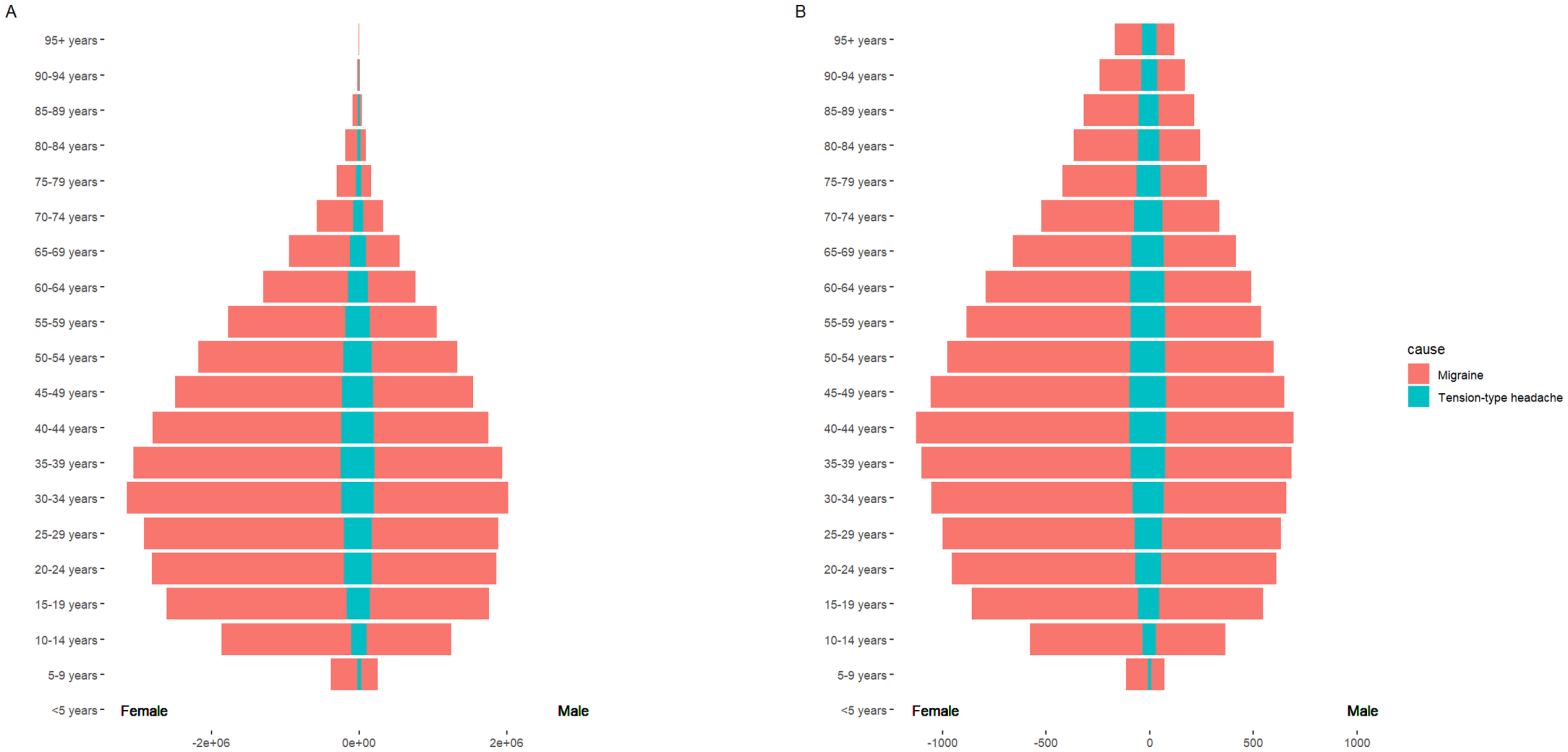
The pyramid model of DALYs of headache disorders. Sex-differentiated (male and female) age-specific distribution of the cause of headache disorders. **(A)** The graph shows the DALYs in terms of case numbers; **(B)** the graph shows the DALYs in terms of the age-standardized rate (ASR).

**Figure 2:**
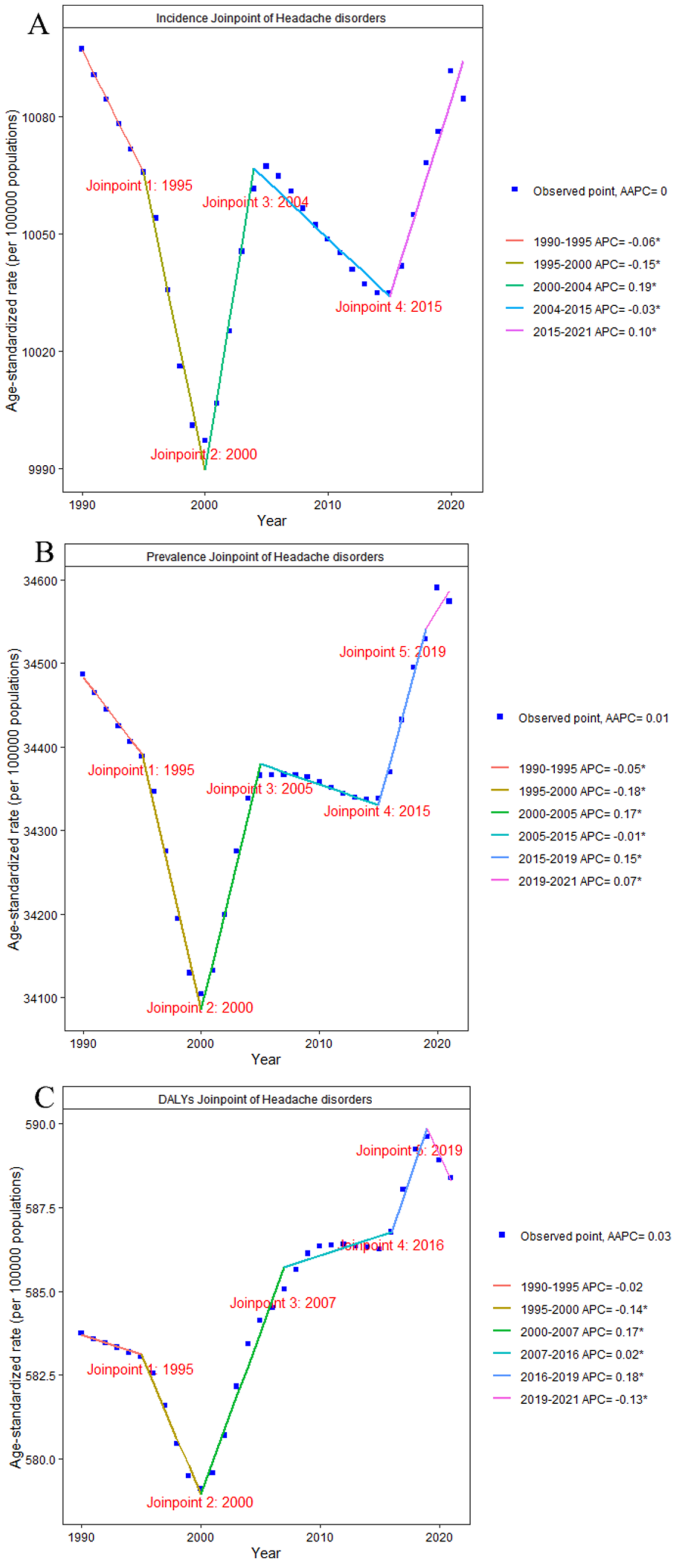
Joinpoint of headache disorders. **(A)** changes over time based on the incidence model; **(B)** changes over time based on the prevalence model; **(C)** changes over time based on the DALYs model. AAPC, average annual percent change; APC, annual percent change; DALYs, disability-adjusted life-years.

We identified headache disorders as a highly prevalent neurological condition worldwide. By 2021, the global incidence was estimated at 809.2 million individuals (95% uncertainty interval [UI]: 717.8 to 896.0 million), while the point prevalence was substantially higher at 2.81 billion (95% UI: 2.60 to 3.03 billion), underscoring the chronic nature of these disorders (Supplementary Tables S1, S2). Tension-type headache was the most common subtype (Supplementary Figures S1, S2), affecting 2.01 billion individuals (95% UI: 1.78 to 2.27 billion; Supplementary Table S2). However, migraine was the predominant driver of disability (Figure 1), accounting for 43.4 million (95% UI: 6.7 to 95.1 million) of the total 48.0 million (95% UI: 9.8 to 100.7 million) DALYs (Supplementary Table S3), revealing a clear dissociation between the high incidence and prevalence of tension-type headache and the high DALYs burden driven by migraine. Our analysis found that the upward global trend since 1990 was not constant, but was characterized by distinct periods of fluctuation. Over the past 32 years, the age-standardized incidence rates (ASIR), prevalence rates, and DALYs for headache disorders have shown an overall upward trend (EAPCs of ASIR = 0.46, age-standardized prevalence rate (ASPR) = 0.56, and age-standardized disability rate (ASDR) = 0.60; Supplementary Tables S1–S3), with considerable fluctuations. Between 1995 and 2000, ASIR, prevalence rates, and DALYs for headache disorders declined (ASIR: APC = –0.15, ASPR: APC = –0.18, ASDR: APC = –0.14), followed by a significant increase from 2000 to 2005 (ASIR: APC = 0.19, ASPR: APC = 0.17, ASDR: APC = 0.17; Figure 2). For migraine, the ASIR, ASPR, and DALYs showed a downward trend before 2000 and an overall upward trend afterward, with two notable periods of increase: 2000–2005 (ASIR: APC = 0.28, ASPR: APC = 0.21, and ASDR: APC = 0.19) and 2015–2019 (ASIR: APC = 0.19, ASPR: APC = 0.22, and ASDR: APC = 0.19; Supplementary Figure S3). Conversely, the ASDR for tension-type headache exhibited an overall downward trend, particularly before 2016, with a trend toward recovery after 2016 (ASDR: APC = 0.08; Supplementary Figure S4).

Females displayed a slightly higher incidence and prevalence of headache disorders than males (Supplementary Figures S1, S2). However, the DALYs for females are significantly higher at 724.76 per 100,000 (95% UI: 137.57 to 1534.57) compared to males at 453.22 per 100,000 (95% UI: 97.50 to 959.01; Figure 1; Supplementary Table S3). The burden of DALYs was concentrated predominantly within the age group of 15–65 years (Figure 1), indicating that headache disorders impose their greatest societal impact during the most economically productive life stages.

### 3.2 Regional and national burden

High-income regions bore the highest rates but experienced the slowest growth, whereas Sub-Saharan Africa showed the most rapid increase in disability burden. Specifically, the highest ASIR was observed in the high-income regions, at 506.31 per 100,000 (95% UI: 999.67 to 13,898.16), despite having the lowest EAPC of ASIR from 1990 to 2021, at 0.16 (Supplementary Table S1). Sub-Saharan Africa had the lowest ASDR at 512.88 per 100,000 (95% UI: 119.10 to 1,061.57); however, the highest EAPC of ASDR at 1.07 from 1990 to 2021 (Supplementary Table S2).

**Figure 3:**
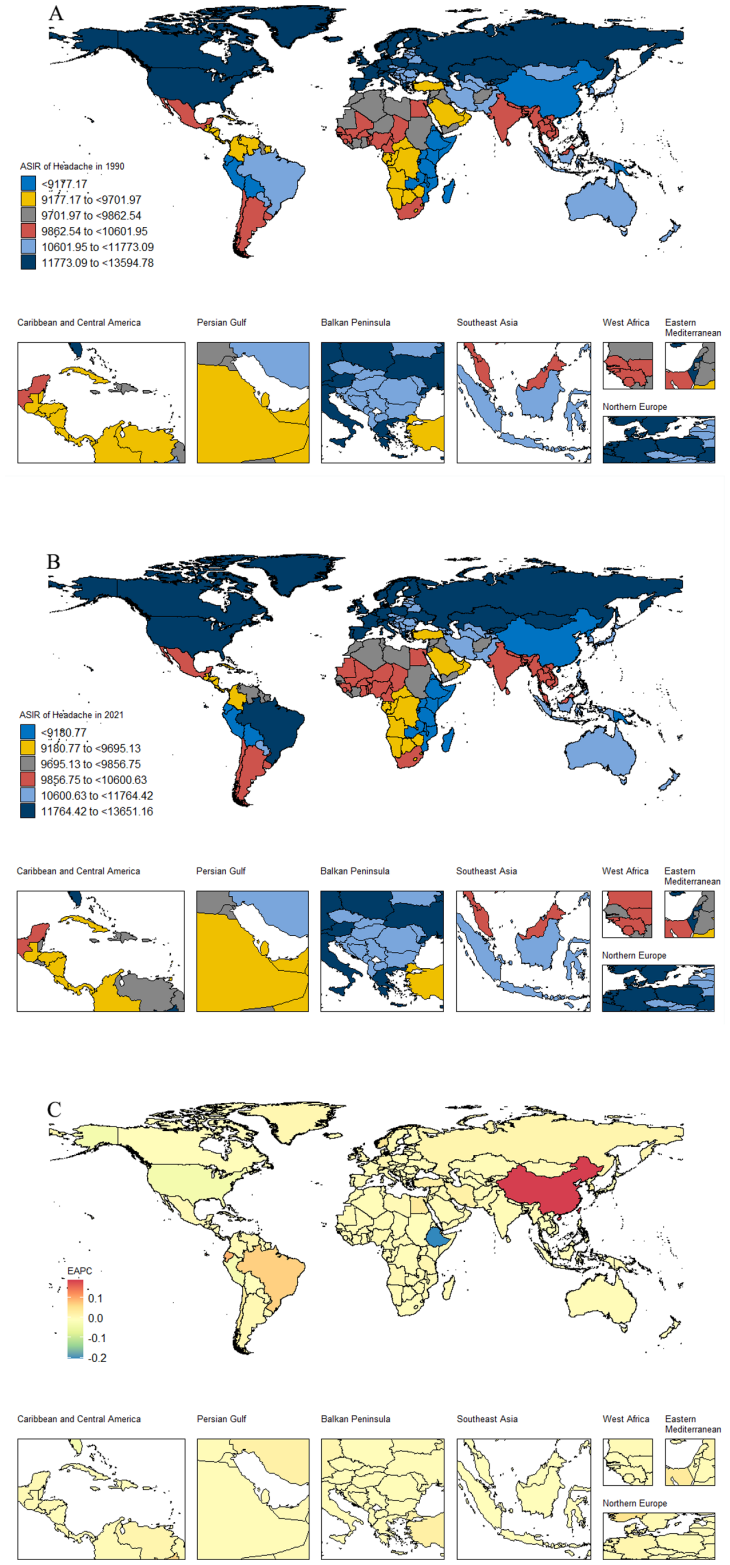
Global distribution maps of the ASIR burden of headache disorders. **(A)** the incidence in terms of the ASR in 1990; **(B)** the incidence in terms of the ASR in 2021; **(C)** the incidence in terms of the EAPC from 1990 to 2021. ASR, age-standardized rate; DALYs, disability-adjusted life-years; EAPC, estimated average percentage change.

The national-level burden exhibited substantial geographical heterogeneity (Figure 3; Supplementary Figures S5–S13). China presented a unique profile, characterized by lower ASR yet a substantial absolute case burden due to its vast population. Notably, China demonstrated a clear increasing trend in headache ASIR and ASPR from 1990 to 2021, contrasting with the stable or declining trends in most other countries. This pattern of high absolute burden and increasing rates was consistent for tension-type headache (Supplementary Figures S10–S12, S15). Peru emerged as another critical case for migraine. Despite a low ASR, the country experienced one of the most significant increases in burden during the study period (Supplementary Figures S7–S9). Although this rise has not yet placed Peru among the highest-burden countries in absolute terms (Supplementary Figure S14), it signals a growing health challenge requiring attention.


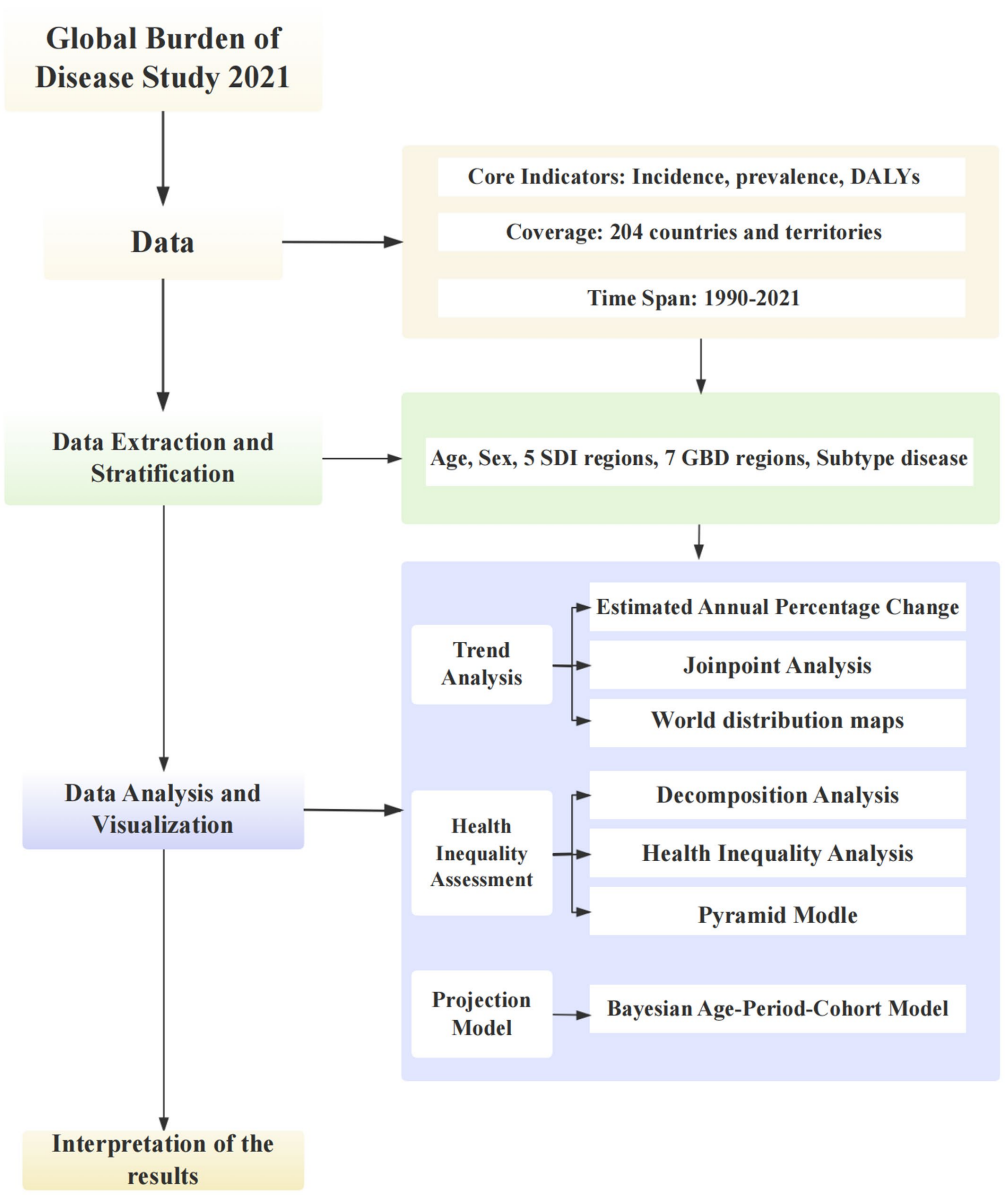


### 3.3 Prediction

**Figure 4:**
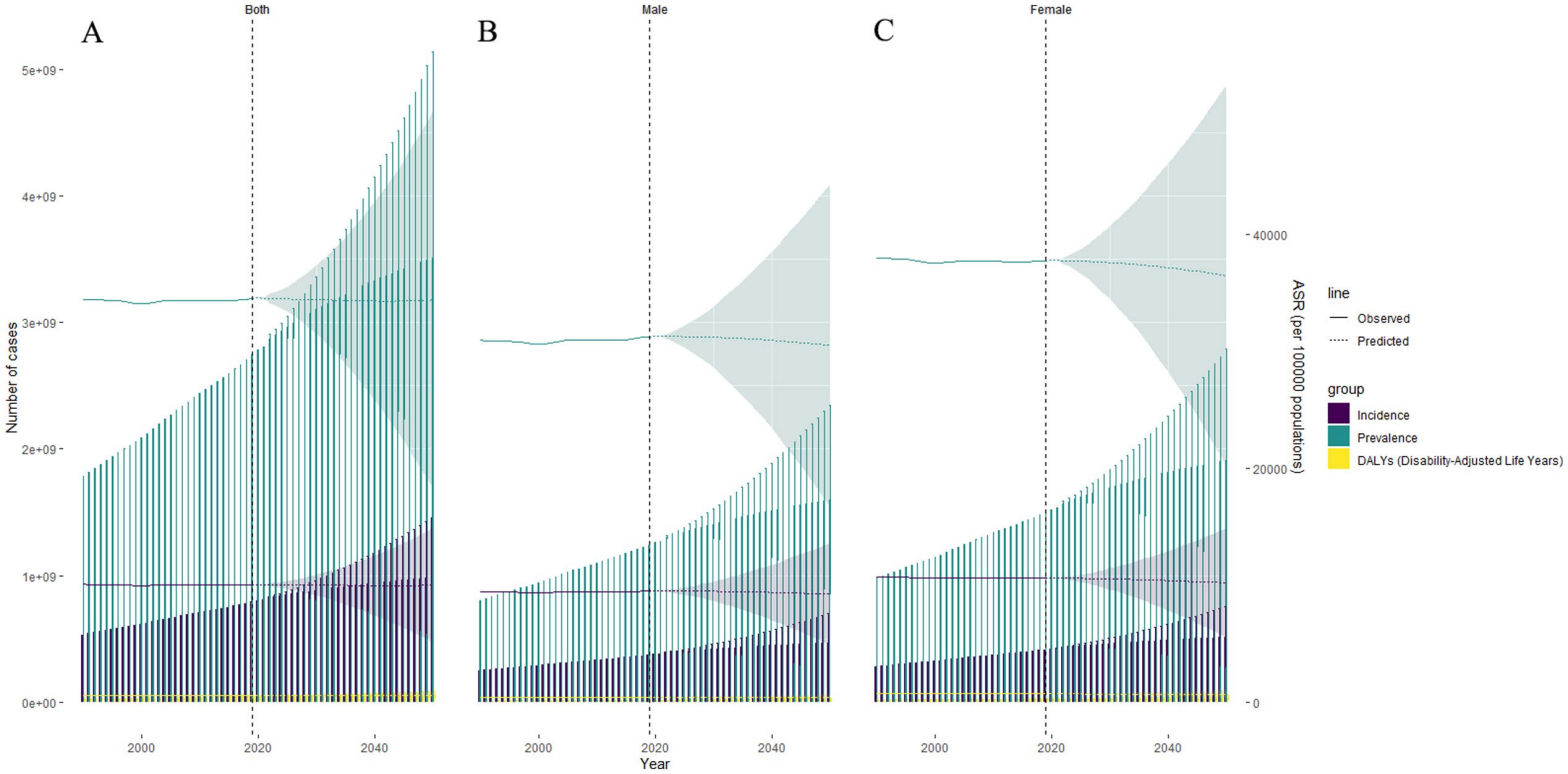
BAPC of headache disorders. **(A)** both sexes; **(B)** male; **(C)** female. BAPC, Bayesian Age-Period-Cohort; ASR, age-standardized rate.

The incidence and prevalence of headache disorders are predicted to continue to increase (Figure 4; Supplementary Figures S16–S17). By 2050, the global prevalence of headache disorders will reach 3.51 billion individuals (95% UI: 1.88 to 5.13 billion), with migraines affecting 1.43 billion individuals (95% UI: 0.74 to 2.12 billion) and tension-type headaches affecting 2.54 billion individuals (95% UI: 1.32 to 3.75 billion). The incidence of tension-type headaches is expected to increase significantly, with a projected global annual incidence of 888.9 million cases (95% UI: 464.2 million to 1.13 billion) by 2050.

### 3.4 Health inequality

Decomposition analysis indicate that regardless of the SDI level, the prevalence of headache disorders is primarily associated with population size (Supplementary Figure 18). The proportional impacts of population size on the global incidence, prevalence, and DALYs of headache disorders were 105.39, 100.11, and 98.99%, respectively (Supplementary Table S4). The results for migraine and tension-type headaches are shown separately in Supplementary Figures S19, S20.

**Figure 5:**
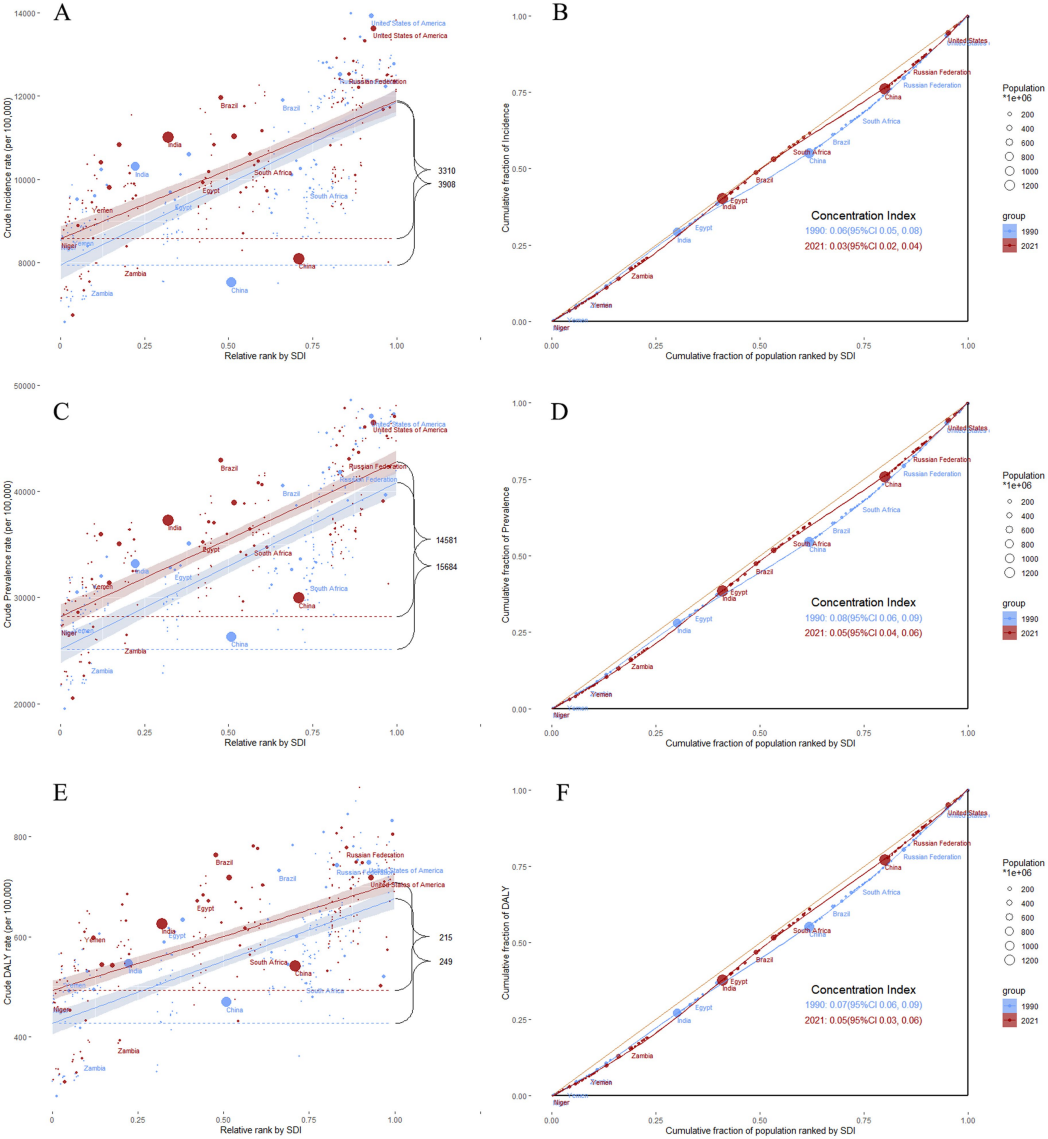
Health inequalities analysis of headache disorders. **(A)** slope indices of inequality based on the incidence of headache disorders in 1990 and 2021 (the numbers adjacent to the brackets indicate the slopes); **(B)** concentration indices of inequality based on the incidence of headache disorders in 1990 and 2021; **(C)** slope indices of inequality based on the prevalence of headache disorders in 1990 and 2021 (the numbers adjacent to the brackets indicate the slopes); **(D)** concentration indices of inequality based on the prevalence of headache disorders in 1990 and 2021; **(E)** slope indices of inequality based on the DALYs of headache disorders in 1990 and 2021 (the numbers adjacent to the brackets indicate the slopes); **(F)** concentration indices of inequality based on the DALYs of headache disorders in 1990 and 2021. In these panels, each country or region is represented by a solid dot, with larger dots indicating a higher population. SDI, Socio-demographic Index; ASR, age-standardized rate.

The slope, intercept, and concentration indices of inequality for headache disorders in 1990 and 2021 are presented in Supplementary Table S5. Health inequality analysis indices revealed a consistent trend toward greater health equity over the study period. The slope indicies and concentration indices showed a substantial narrowing of the disparity in headache burden between low- and high-SDI countries. From 1990 to 2021, the slopes for incidence, prevalence, and DALYs all decreased: from 3907.88 (95% CI: 3398.9 to 4416.87) to 3309.71 (95% CI: 2855.38 to 3764.04), 15683.76 (95% CI: 13785.4 to 17582.12) to 14581.02 (95% CI: 12943.53 to 16218.52), and 248.66 (95% CI: 210.58 to 286.75) to 215.19 (95% CI: 180.64 to 249.74), respectively (Supplementary Table S5). Additionally, the concentration indices for the incidence, prevalence, and DALYs exhibited a downward trend from 1990 to 2021, approaching zero (Figure 5). This trend suggests a diminishing disparity in the burden of headache disorders across countries with varying SDI scores. Specifically, the concentration indices declined from 0.064 (95% CI: 0.0509 to 0.0771) to 0.0319 (95% CI: 0.0196 to 0.0441) for incidence; from 0.0774 (95% CI: 0.0649 to 0.0898) to 0.0484 (95% CI: 0.037 to 0.0598) for prevalence; and from 0.0729 (95% CI: 0.0599 to 0.086) to 0.0466 (95% CI: 0.0343 to 0.0589) for DALYs (Supplementary Table S5). However, this reduction in relative inequality coincided with a rise in absolute inequality. An increase in the intercept values implied that when the SDI was at its lowest level, the burden of disease increased. From 1990 to 2021, the intercepts for incidence, prevalence, and DALYs steadily increased: from 7945.61 (95% CI: 7625.46 to 8265.77) to 8578.14 (95% CI: 8293 to 8863.28), 25136.05 (95% CI: 23941.96 to 26330.15) to 28202.88 (95% CI: 27175.2 to 29230.56), and 427.23 (95% CI: 403.28 to 451.19) to 492.89 (95% CI: 471.21 to 514.58), respectively (Supplementary Table S5). The results for migraine and tension-type headaches are presented in Supplementary Figures S21, S22.

## 4 Discussion

In 2023, WHO emphasizes the importance of paying attention to headache conditions. It highlights the slogan “Pay attention to headaches and keep them at bay—We are always in action,” signaling a strong commitment to addressing the issue (12). Headache, one of the most common and disabling chronic pain conditions, is a key component of this action plan. A deeper understanding of the disease burden, epidemiological trends, and factors driving these trends in headache disorders is crucial for developing public health policies. This comprehensive analysis found that tension-type headache is the most prevalent subtype, while migraine is the primary driver of disability, with a significant disproportionate burden on women and the working-age population. Critically, our health inequality analysis revealed a trend toward reduced relative disparities between high- and low-SDI regions, yet an increase in the absolute burden at the lowest SDI levels.

Globally, over the past 32 years, the incidence and prevalence of headache disorders, including migraine and tension-type headaches, have shown fluctuating trends alongside an overall upward trajectory, with predictions suggesting continued growth. This increasingly severe public health issue requires further attention. Several reasons are responsible for this phenomenon. Firstly, as the global population grows, the total number of individuals suffering from headache disorders increases. Our decomposition analysis shows that the proportionate impact of population size on the global incidence, prevalence, and DALYs of headache disorders is 105.39, 100.11, and 98.99%, respectively. Secondly, changing environmental and social factors are important contributing factors. Modern lifestyle factors such as stress (9), insufficient sleep (13–15), and alterations in dietary habits (16) may contribute to an increased incidence of headache disorders. High consumption of alcohol, salt, pork, and poultry intake is associated with higher multisite chronic pain scores (16). Environmental factors, including pollution (17, 18) and climate change (18), can affect the incidence of headache disorders. Globalization and urbanization have led to notable lifestyle changes, increasing work-related stress, sleep deprivation, air pollution, and climate change. Tirdly, advancements in medical technology and a heightened awareness of headache disorders have improved the accuracy of diagnosis and reporting, which may have led to increased statistical data. Improving socioeconomic conditions may afford better access to healthcare resources, increasing the number of diagnosed and treated cases. Increased public awareness of headache disorders may lead more people to seek medical assistance, increasing the number of reported cases. Changes in the healthcare system, such as a growing emphasis on chronic pain management, may contribute to higher diagnosis and reporting rates. Finally, while the GBD methodology is standardized, methodological shifts in case definitions, data processing, and modeling algorithms over the different GBD iterations could contribute to some of the observed trends, making it challenging to disentangle true change from improved measurement.

The burden of headache disorders varies across countries and regions, highlighting the need for tailored health policies. An analysis of health inequities reveals that the gap between low and high SDI regions is narrowing and the absolute burden of the lowest SDI levels is increasing. Those trend may result from the increasing emphasis on chronic pain management in areas with a low SDI, which has raised public awareness of headache disorders and led to increased diagnosis and reporting. Additionally, adverse environmental conditions and greater lifestyle stress in these regions may contribute to this trend. In regions with a high SDI, the burden remains substantial, potentially because public health policies have focused primarily on diagnosis and reporting with insufficient policy support for treatment. Additionally, the chronic and often lifelong nature of headache disorders and the lack of effective options may contribute to this burden. Our study not only underscores the urgency of developing targeted public health strategies and interventions to address the growing burden of headache disorders but also highlights the need for research into effective treatment options to control the onset of headaches.

Apart from regional disparities, sex and age were significant factors. Our research found that migraines disproportionately affect women, while tension-type headaches are more prevalent in men. This well-documented pattern underscores the profound influence of sex-specific biological (e.g., hormonal) (19) and psychosocial factors (20). Moreover, research on tension-type headaches is relatively scarce, indicating the need for further investigations. Our study identified a higher incidence of headaches among individuals aged 15–65, which may be associated with longer hours of sedentary work (21), increased screen time (22, 23), and greater academic and work-related pressure in this age group.

This study acknowledges its limitations, including its reliance on the GBD database and the potential uncertainty of the modeled data resulting from data scarcity, a paramount limitation particularly in LMICs. The wide UI surrounding our 2050 projections must be emphasized. These projections are not deterministic forecasts but rather plausible scenarios based on the continuation of past and current trends. They do not account for unpredictable future breakthroughs in treatment (e.g., novel therapeutics like PACAP or amylin antibodies) or profound societal shifts (e.g., widespread adoption of AI altering work environments and stress patterns). Despite these limitations, it provides valuable insights into headache research and public health.

Our findings underscore the increasing global burden of headache disorders and emphasize the urgent need to implement comprehensive public health strategies. These strategies include targeted interventions and resource allocation to mitigate this burden. For example, integrate headache care into primary health systems by training general practitioners under neurologist supervision to enhance access and reduce specialist burden. Implement public health campaigns to promote lifestyle modifications. Enact workplace policies, such as ergonomic and screen-time guidelines, targeting the high-burden working-age population. Future research should focus on elucidating the drivers of this increasing burden, particularly lifestyle and environmental risk factors. Additionally, rigorous evaluations of the effectiveness of the proposed interventions, such as educational campaigns and the integration of headache management into primary care, are necessary. This evaluation will inform evidence-based public health strategies to address the growing burden of headache disorders.

## 5 Conclusion

Our systematic analysis of the GBD 2021 highlights the substantial and growing burden of headache disorders, including migraines and tension-type headaches, on global, regional, and national scales from 1990 to 2021. Predictions for 2050 underscore the potentail need for targeted public health strategies and interventions to mitigate this increasing burden. These findings necessitate the development of evidence-based interventions and policies to alleviate the impact of headache disorders on individuals and society.

## Data Availability

Publicly available datasets were analyzed in this study. This data can be found at: https://vizhub.healthdata.org/gbd-results/.

## Supplementary material

SUPPLEMENTARY FIGURE S1 The pyramid model of incidence of headache disorders. Sex-differentiated (male and female) age-specific distribution of the cause of headache disorders. **(A)** The graph shows the incidence in terms of case numbers; **(B)** the graph shows the incidence in terms of the age-standardized rate (ASR).

SUPPLEMENTARY FIGURE S2 The pyramid model of prevalence of headache disorders. Sex-differentiated (male and female) age-specific distribution of the cause of headache disorders. **(A)** The graph shows the prevalence in terms of case numbers; **(B)** the graph shows the prevalence in terms of the age-standardized rate (ASR).

SUPPLEMENTARY FIGURE S3 Joinpoint of migraine. **(A)** Changes over time based on the incidence model; **(B)** changes over time based on the prevalence model; **(C)** changes over time based on the DALYs model. AAPC, average annual percent change; APC, annual percent change; DALYs, disability-adjusted life-years.

SUPPLEMENTARY FIGURE S4 Joinpoint of tension-type headache. **(A)** Changes over time based on the incidence model; **(B)** changes over time based on the prevalence model; **(C)** changes over time based on the DALYs model. AAPC, average annual percent change; APC, annual percent change; DALYs, disability-adjusted life-years.

SUPPLEMENTARY FIGURE S5 Global distribution maps of the ASPR burden from headache disorders. **(A)** The prevalence in terms of the ASR in 1990; **(B)** the prevalence in terms of the ASR in 2021; **(C)** the prevalence in terms of the EAPC from 1990 to 2021. ASR, age-standardized rate; EAPC, estimated average percentage change.

SUPPLEMENTARY FIGURE S6 Global distribution maps of the ASDR burden from headache disorders. **(A)** the DALYs in terms of the ASR in 1990; **(B)** the DALYs in terms of the ASR in 2021; **(C)** the DALYs in terms of the EAPC from 1990 to 2021. ASR, age-standardized rate; EAPC, estimated average percentage change; DALYs, disability-adjusted life-years.

SUPPLEMENTARY FIGURE S7 Global distribution maps of the ASIR burden from migraine. **(A)** The incidence in terms of the ASR in 1990; **(B)** the incidence in terms of the ASR in 2021; **(C)** the incidence in terms of the EAPC from 1990 to 2021. ASR, age-standardized rate; EAPC, estimated average percentage change.

SUPPLEMENTARY FIGURE S8 Global distribution maps of the ASPR burden from migraine. **(A)** The prevalence in terms of the ASR in 1990; **(B)** the prevalence in terms of the ASR in 2021; **(C)** the prevalence in terms of the EAPC from 1990 to 2021. ASR, age-standardized rate; EAPC, estimated average percentage change.

SUPPLEMENTARY FIGURE S9 Global distribution maps of the ASDR burden from migraine. **(A)** The DALYs in terms of the ASR in 1990; **(B)** the DALYs in terms of the ASR in 2021; **(C)** the DALYs in terms of the EAPC from 1990 to 2021. ASR, age-standardized rate; EAPC, estimated average percentage change; DALYs, disability-adjusted life-years.

SUPPLEMENTARY FIGURE S10 Global distribution maps of the ASIR burden from tension-type headache. **(A)** The incidence in terms of the ASR in 1990; **(B)** the incidence in terms of the ASR in 2021; **(C)** the incidence in terms of the EAPC from 1990 to 2021. ASR, age-standardized rate; EAPC, estimated average percentage change.

SUPPLEMENTARY FIGURE S11 Global distribution maps of the ASPR burden from tension-type headache. **(A)** The prevalence in terms of the ASR in 1990; **(B)** the prevalence in terms of the ASR in 2021; **(C)** the prevalence in terms of the EAPC from 1990 to 2021. ASR, age-standardized rate; EAPC, estimated average percentage change.

SUPPLEMENTARY FIGURE S12 Global distribution maps of the ASDR burden from tension-type headache. **(A)** The DALYs in terms of the ASR in 1990; **(B)** the DALYs in terms of the ASR in 2021; **(C)** the DALYs in terms of the EAPC from 1990 to 2021. ASR, age-standardized rate; EAPC, estimated average percentage change; DALYs, disability-adjusted life-years.

SUPPLEMENTARY FIGURE S13 Global distribution maps of the number burden from headache disorders. **(A)** The incidence in terms of the number in 1990; **(B)** the incidence in terms of the number in 2021; **(C)** the prevalence in terms of the number in 1990; **(D)** the prevalence in terms of the number in 2021; **(E)** the DALYs in terms of the number in 1990; **(F)** the DALYs in terms of the number in 2021. ASR, age-standardized rate; EAPC, estimated average percentage change; DALYs, disability-adjusted life-years.

SUPPLEMENTARY FIGURE S14 Global distribution maps of the number burden from migraine. **(A)** The incidence in terms of the number in 1990; **(B)** the incidence in terms of the number in 2021; **(C)** the prevalence in terms of the number in 1990; **(D)** the prevalence in terms of the number in 2021; **(E)** the DALYs in terms of the number in 1990; **(F)** the DALYs in terms of the number in 2021. ASR, age-standardized rate; EAPC, estimated average percentage change; DALYs, disability-adjusted life-years.

SUPPLEMENTARY FIGURE S15 Global distribution maps of the number burden from tension-type headache. **(A)** The incidence in terms of the number in 1990; **(B)** the incidence in terms of the number in 2021; **(C)** the prevalence in terms of the number in 1990; **(D)** the prevalence in terms of the number in 2021; **(E)** the DALYs in terms of the number in 1990; **(F)** the DALYs in terms of the number in 2021. ASR, age-standardized rate; EAPC, estimated average percentage change; DALYs, disability-adjusted life-years.

SUPPLEMENTARY FIGURE S16 BAPC of migraine. **(A)** Both sexes; **(B)** male; **(C)** female. BAPC, Bayesian Age-Period-Cohort; ASR, age-standardized rate.

SUPPLEMENTARY FIGURE S17 BAPC of tension-type headache. **(A)** Both sexes; **(B)** male; **(C)** female. BAPC, Bayesian Age-Period-Cohort; ASR, age-standardized rate.

SUPPLEMENTARY FIGURE S18 The decomposition of Headache disorders. Changes in the burden of headache disorders according to population-level determinants (population growth, aging, and epidemiological changes) from 1990 to 2021 at the global level based on five SDI quintiles. **(A)** DALYs rate; **(B)** incidence rate; **(C)** prevalence rate. In all panels, the black dots represent the overall value of the change based on the contribution of all three components. DALYs, disability-adjusted life-years; GBD, Global Burden of Disease; SDI, Socio-demographic Index.

SUPPLEMENTARY FIGURE S19 The decomposition of migraine. Changes in the burden of migraine according to population-level determinants (population growth, aging, and epidemiological changes) from 1990 to 2021 at the global level based on five SDI quintiles. **(A)** DALYs rate; **(B)** incidence rate; **(C)** prevalence rate. In all panels, the black dots represent the overall value of the change based on the contribution of all three components. DALYs, disability-adjusted life-years; GBD, Global Burden of Disease; SDI, Socio-demographic Index.

SUPPLEMENTARY FIGURE S20 The decomposition of tension-type headache. Changes in the burden of tension-type headache according to population-level determinants (population growth, aging, and epidemiological changes) from 1990 to 2021 at the global level based on five SDI quintiles. **(A)** DALYs rate; **(B)** incidence rate; **(C)** prevalence rate. In all panels, the black dots represent the overall value of the change based on the contribution of all three components. DALYs, disability-adjusted life-years; GBD, Global Burden of Disease; SDI, Socio-demographic Index.

SUPPLEMENTARY FIGURE S21 Health inequalities analysis of migraine. **(A)** Slope indices of inequality based on the incidence of migraine in 1990 and 2021 (the numbers adjacent to the brackets indicate the slopes); **(B)** concentration indices of inequality based on the incidence of migraine in 1990 and 2021; **(C)** slope indices of inequality based on the prevalence of migraine in 1990 and 2021 (the numbers adjacent to the brackets indicate the slopes); **(D)** concentration indices of inequality based on the prevalence of migraine in 1990 and 2021; **(E)** slope indices of inequality based on the DALYs of migraine in 1990 and 2021 (the numbers adjacent to the brackets indicate the slopes); **(F)** concentration indices of inequality based on the DALYs of migraine in 1990 and 2021. In these panels, each country or region is represented by a solid dot, with larger dots indicating a higher population. SDI, Socio-demographic Index; ASR, age-standardized rate.

SUPPLEMENTARY FIGURE S22 Health inequalities analysis of tension-type headache. **(A)** Slope indices of inequality based on the incidence of tension-type headache in 1990 and 2021 (the numbers adjacent to the brackets indicate the slopes); **(B)** concentration indices of inequality based on the incidence of tension-type headache in 1990 and 2021; **(C)** slope indices of inequality based on the prevalence of tension-type headache in 1990 and 2021 (the numbers adjacent to the brackets indicate the slopes); **(D)** concentration indices of inequality based on the prevalence of tension-type headache in 1990 and 2021; **(E)** slope indices of inequality based on the DALYs of tension-type headache in 1990 and 2021 (the numbers adjacent to the brackets indicate the slopes); **(F)** concentration indices of inequality based on the DALYs of tension-type headache in 1990 and 2021. In these panels, each country or region is represented by a solid dot, with larger dots indicating a higher population. SDI, Socio-demographic Index; ASR, age-standardized rate.
