# Supplementary figures and images for: Global, regional, and national burden of headache disorders, 1990–2021, and projections to 2050: a comprehensive analysis of the global burden of disease study 2021
Source: Front Neurol. 2025 Oct 29;16:1674946. doi: 10.3389/fneur.2025.1674946 (PMC12608083; doi:10.3389/fneur.2025.1674946)

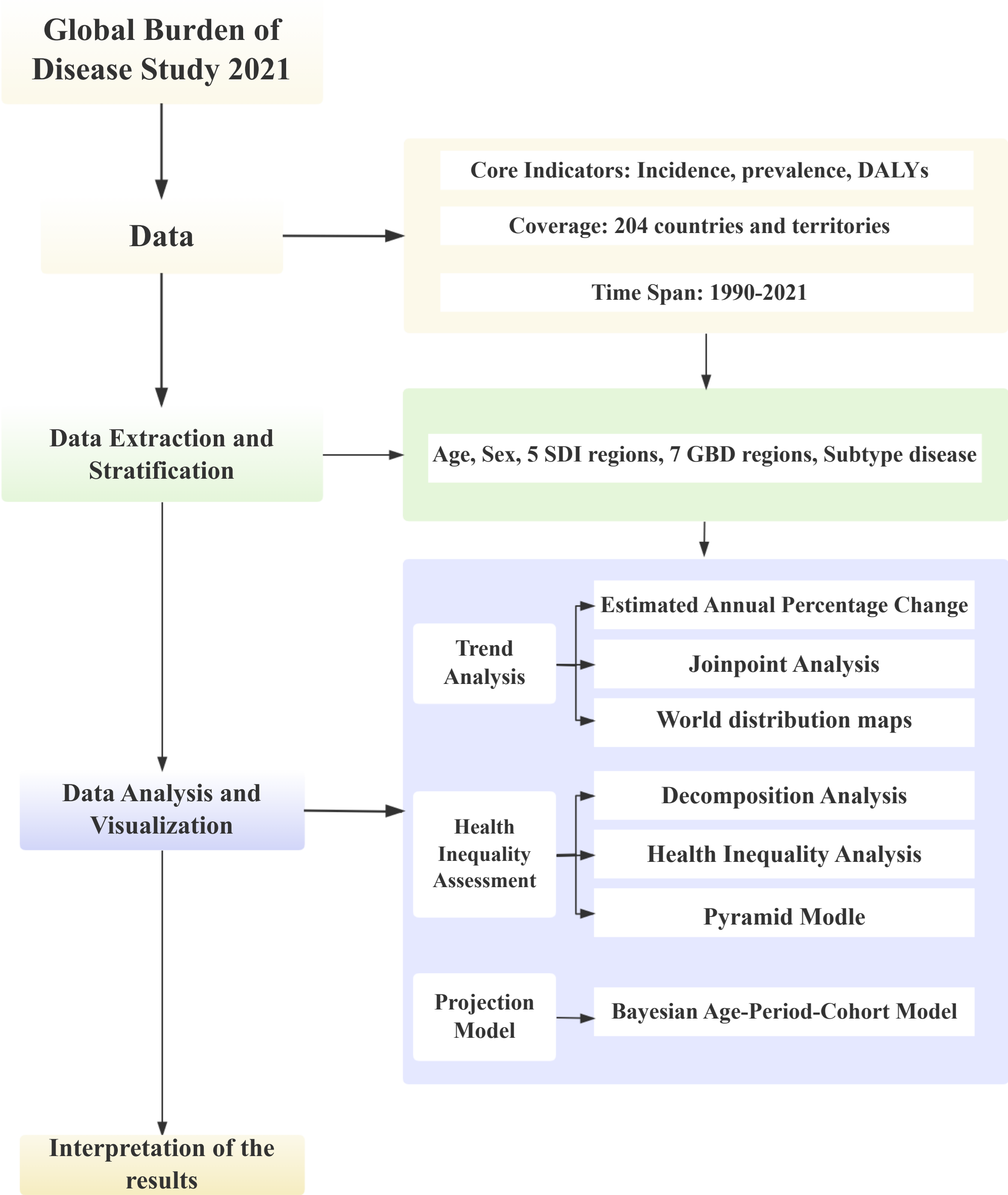

Supplement: Supplementary file 1 [file Data_Sheet_1.zip › Supporting Information/Flowchart.pdf]

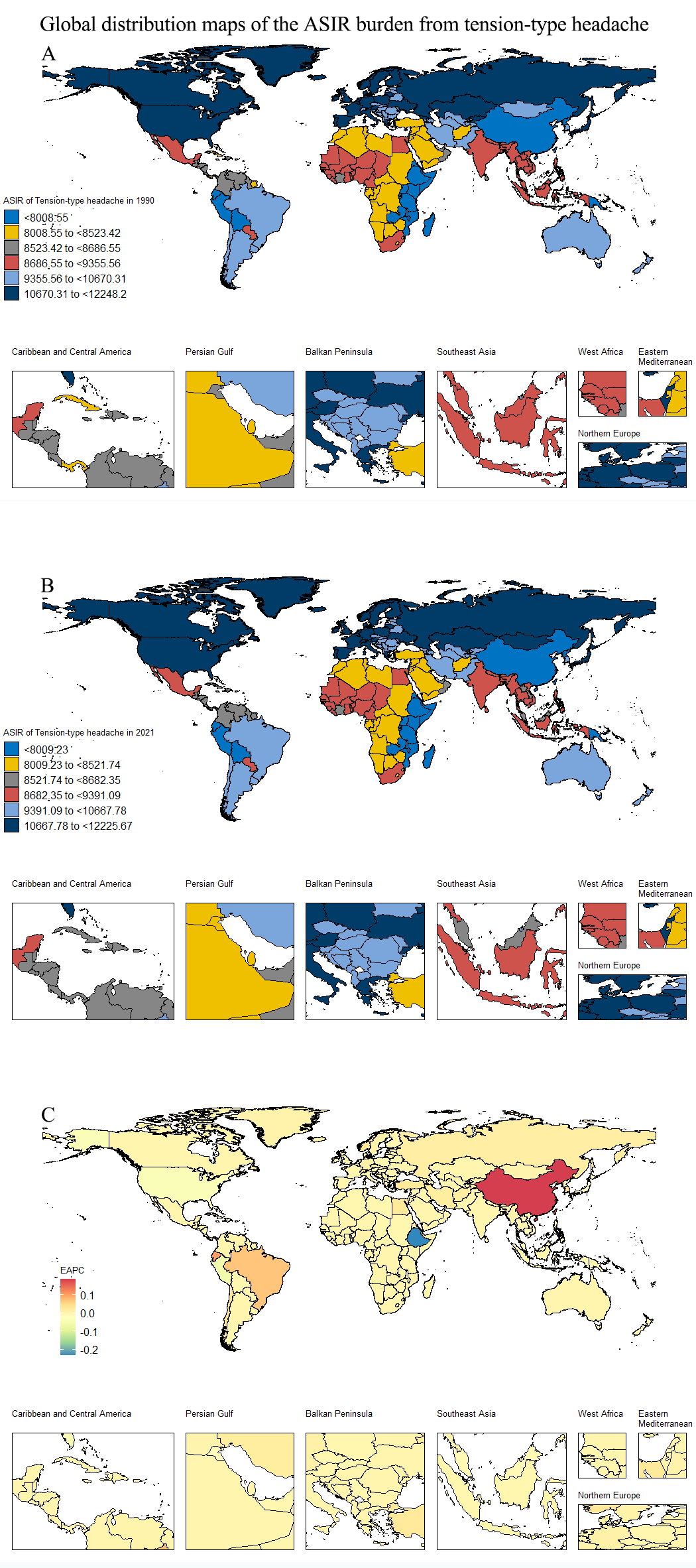

Supplement: Supplementary file 1 [file Data_Sheet_1.zip › Supporting Information/S10_Fig.tif]

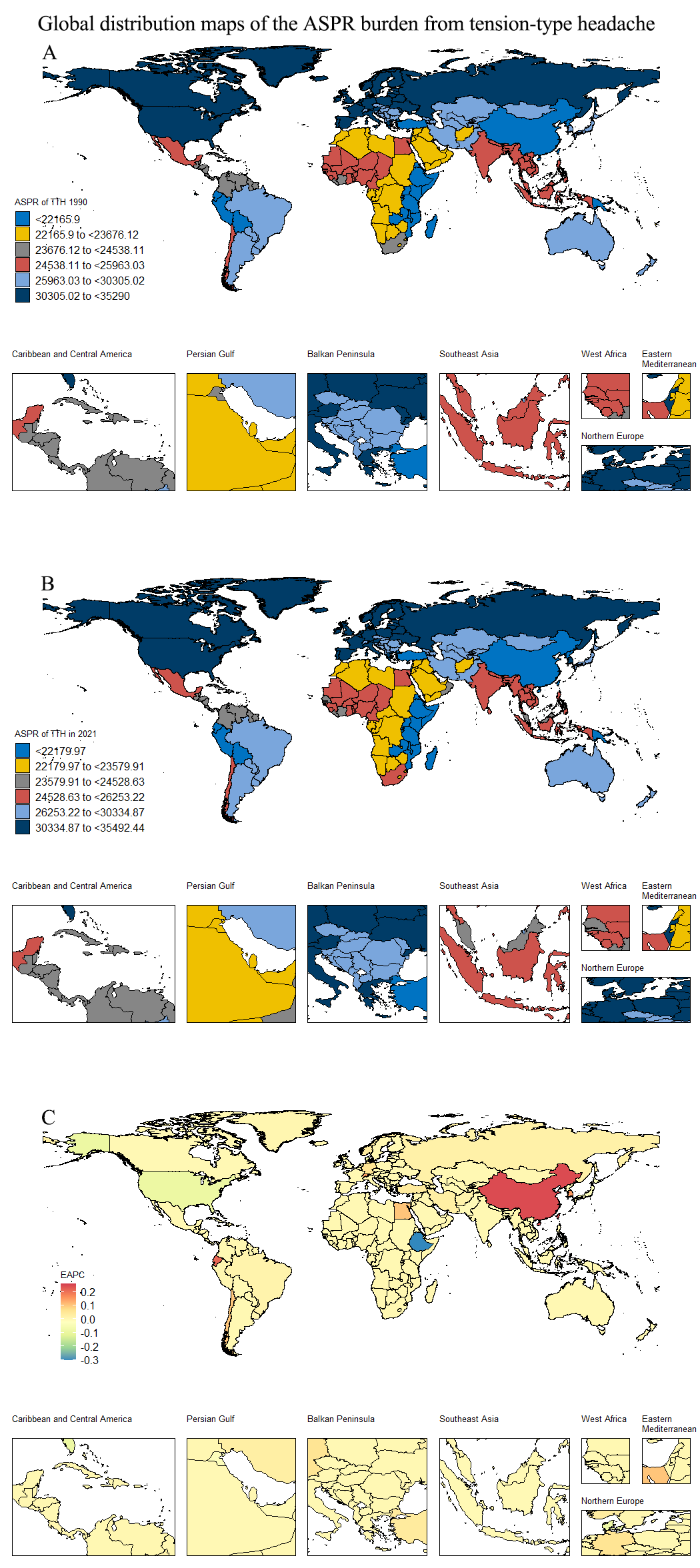

Supplement: Supplementary file 1 [file Data_Sheet_1.zip › Supporting Information/S11_Fig.tif]

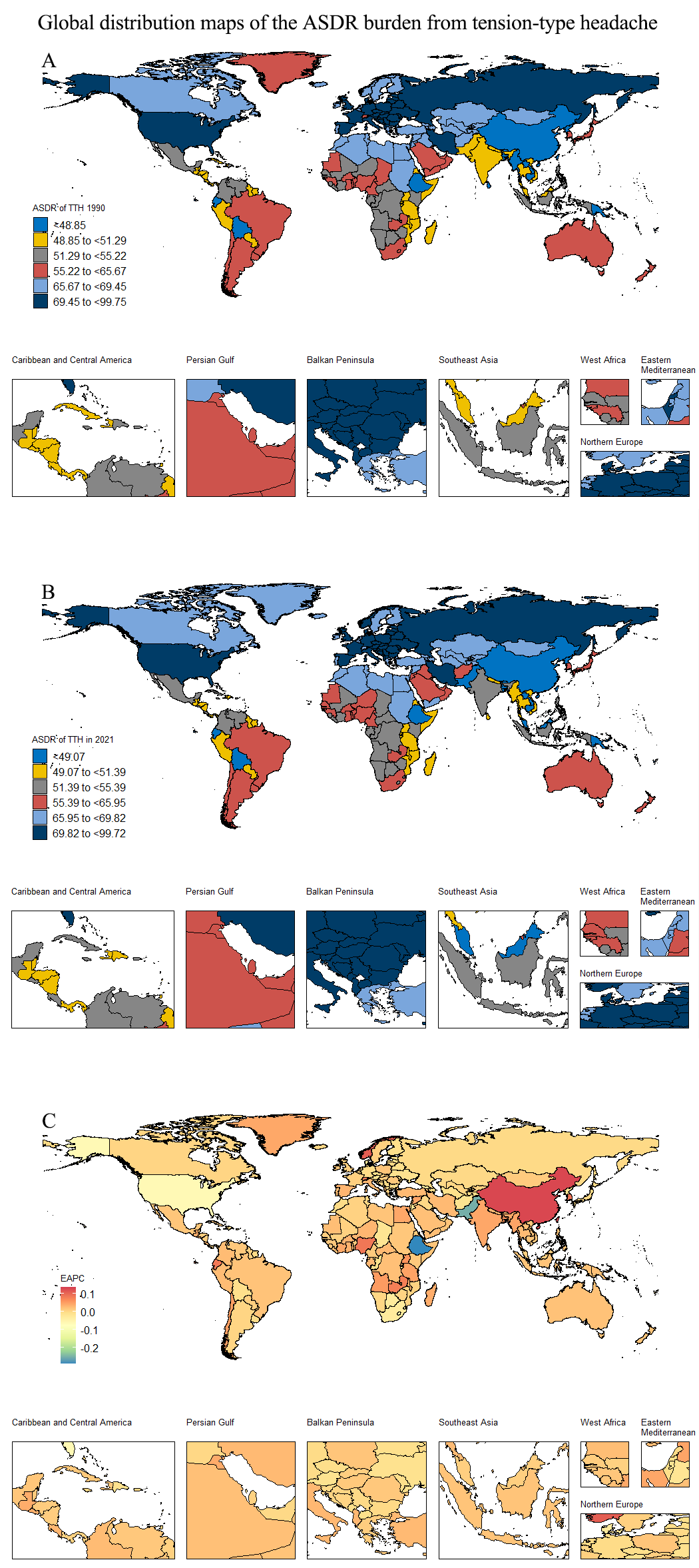

Supplement: Supplementary file 1 [file Data_Sheet_1.zip › Supporting Information/S12_Fig.tif]

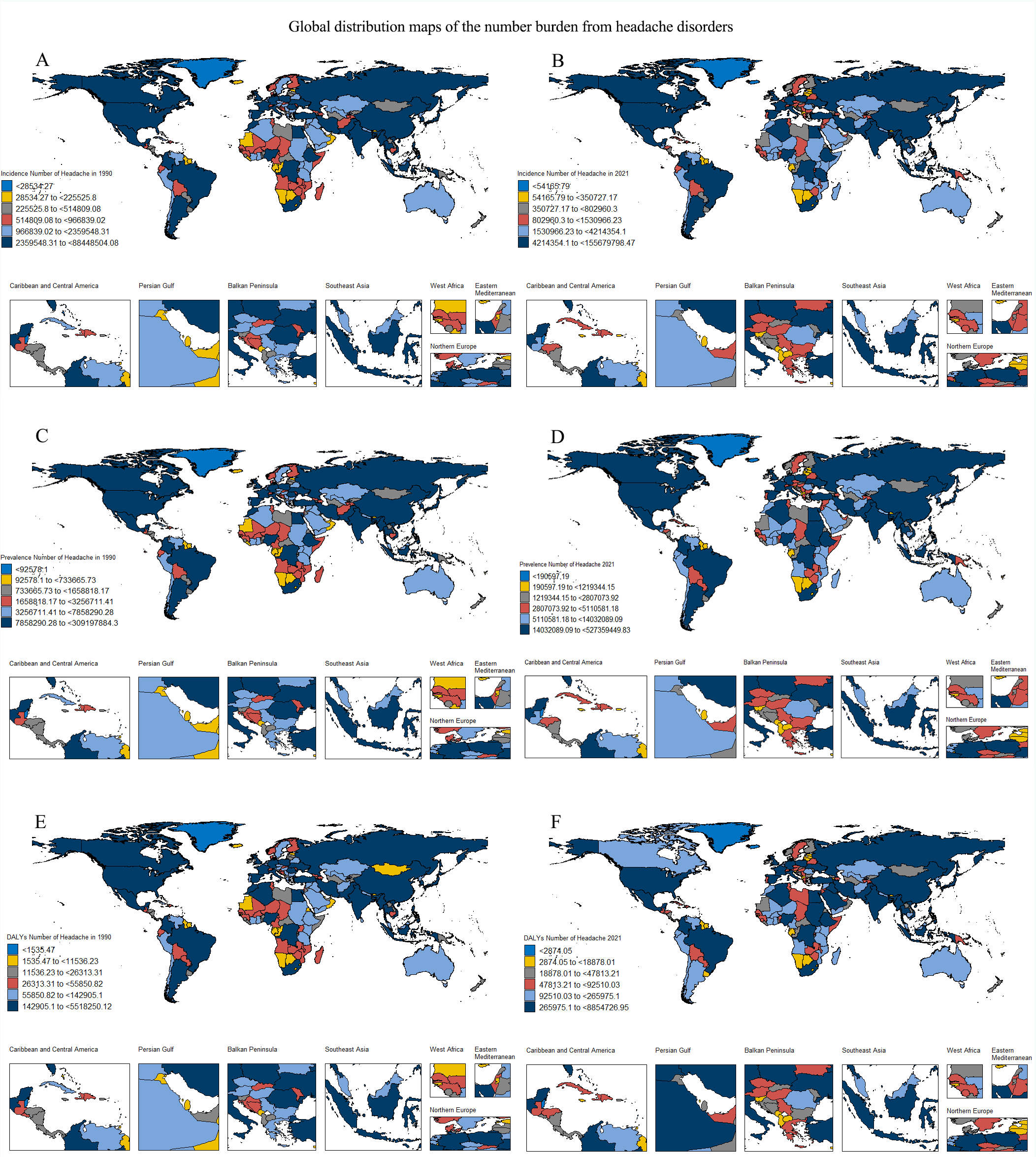

Supplement: Supplementary file 1 [file Data_Sheet_1.zip › Supporting Information/S13_Fig.tif]

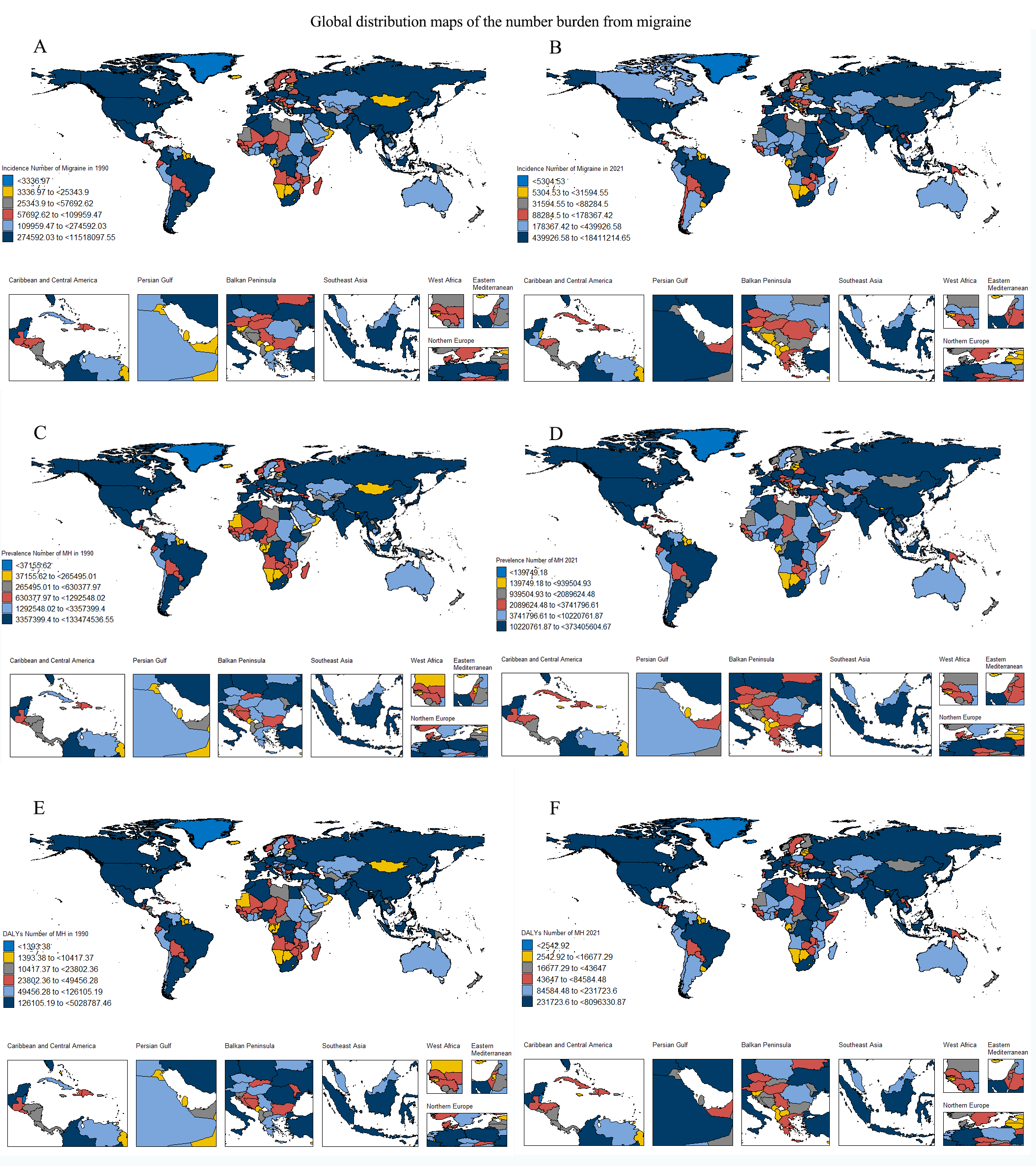

Supplement: Supplementary file 1 [file Data_Sheet_1.zip › Supporting Information/S14_Fig.tif]

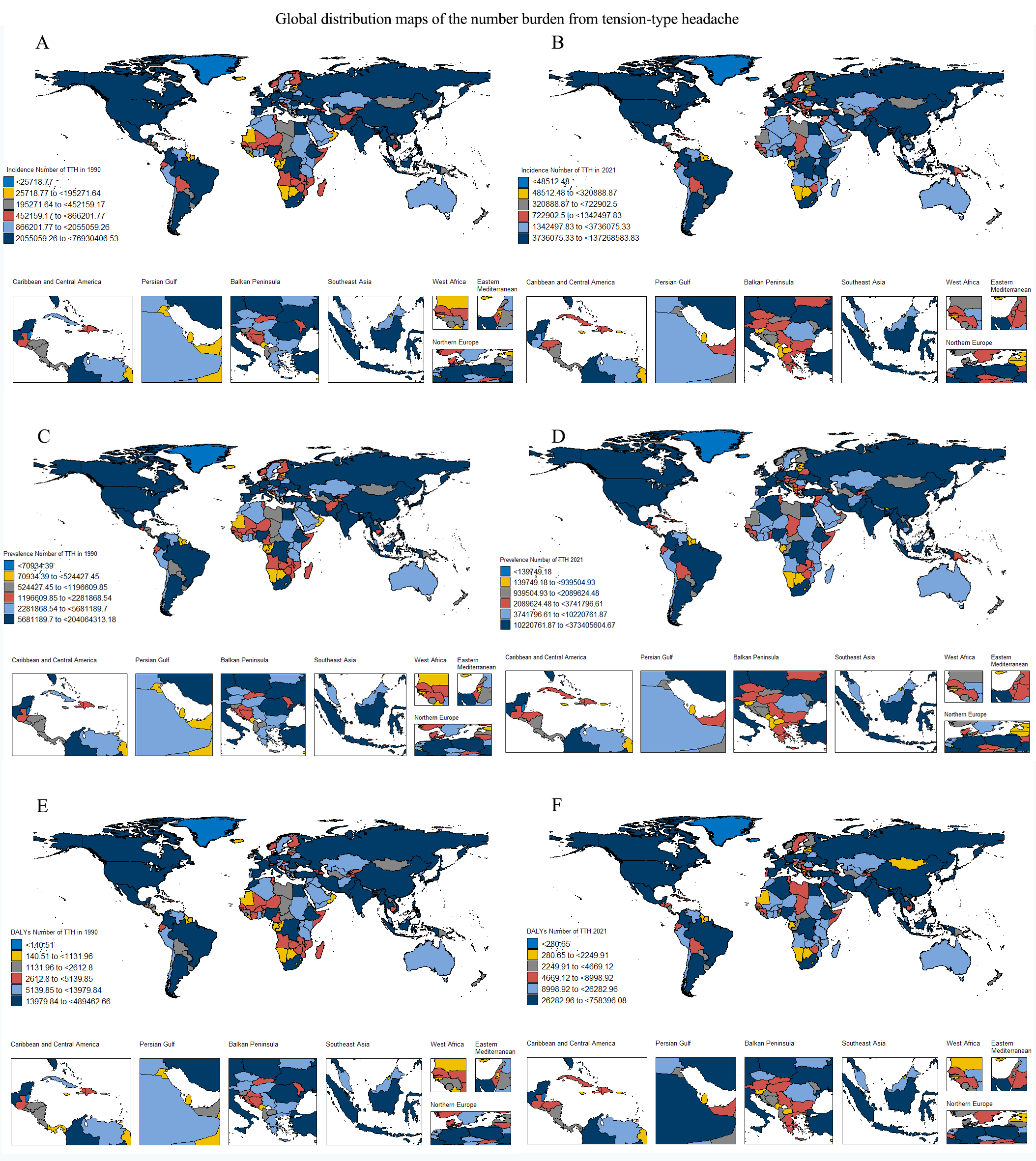

Supplement: Supplementary file 1 [file Data_Sheet_1.zip › Supporting Information/S15_Fig.tif]

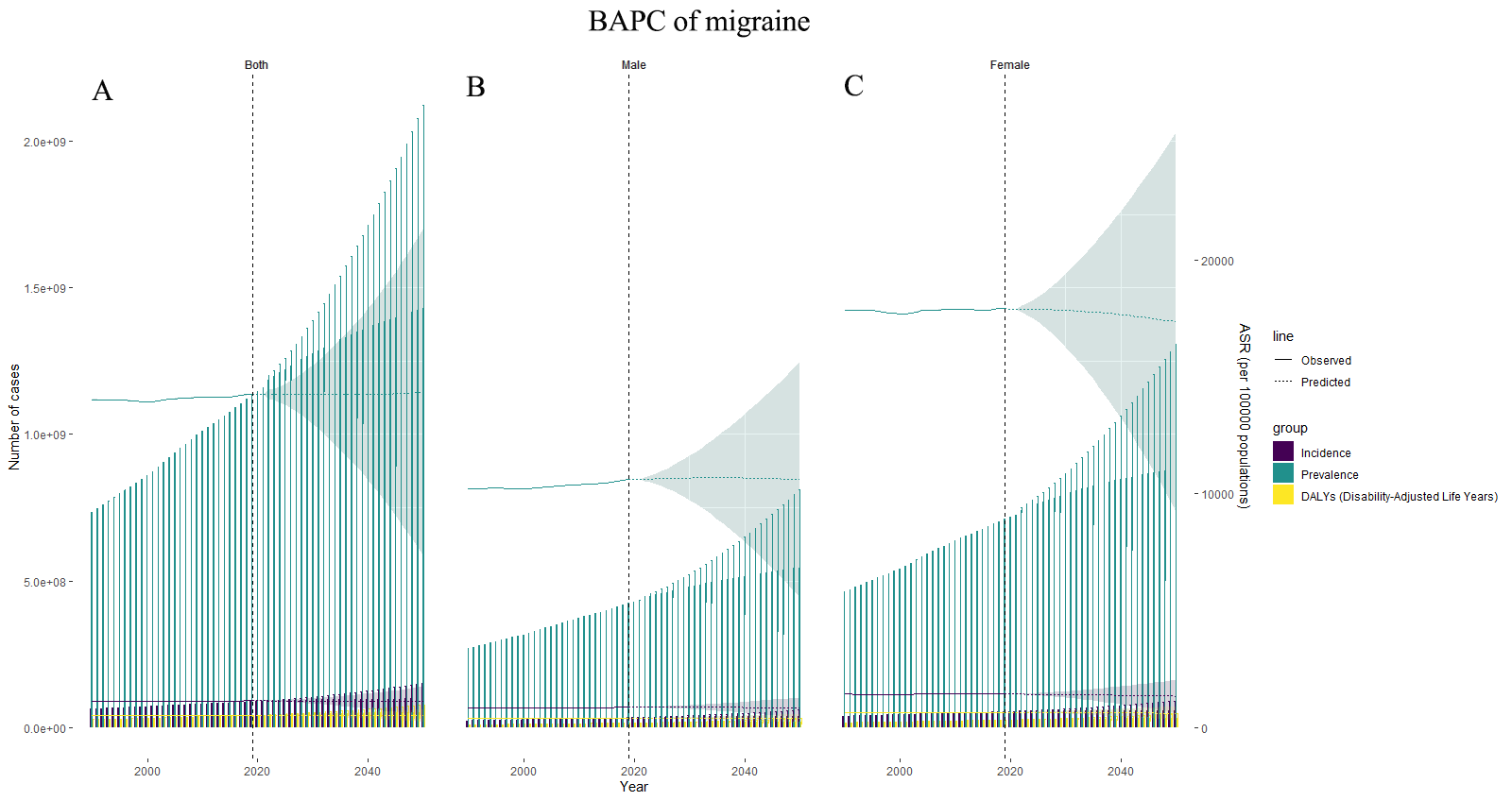

Supplement: Supplementary file 1 [file Data_Sheet_1.zip › Supporting Information/S16_Fig.tif]

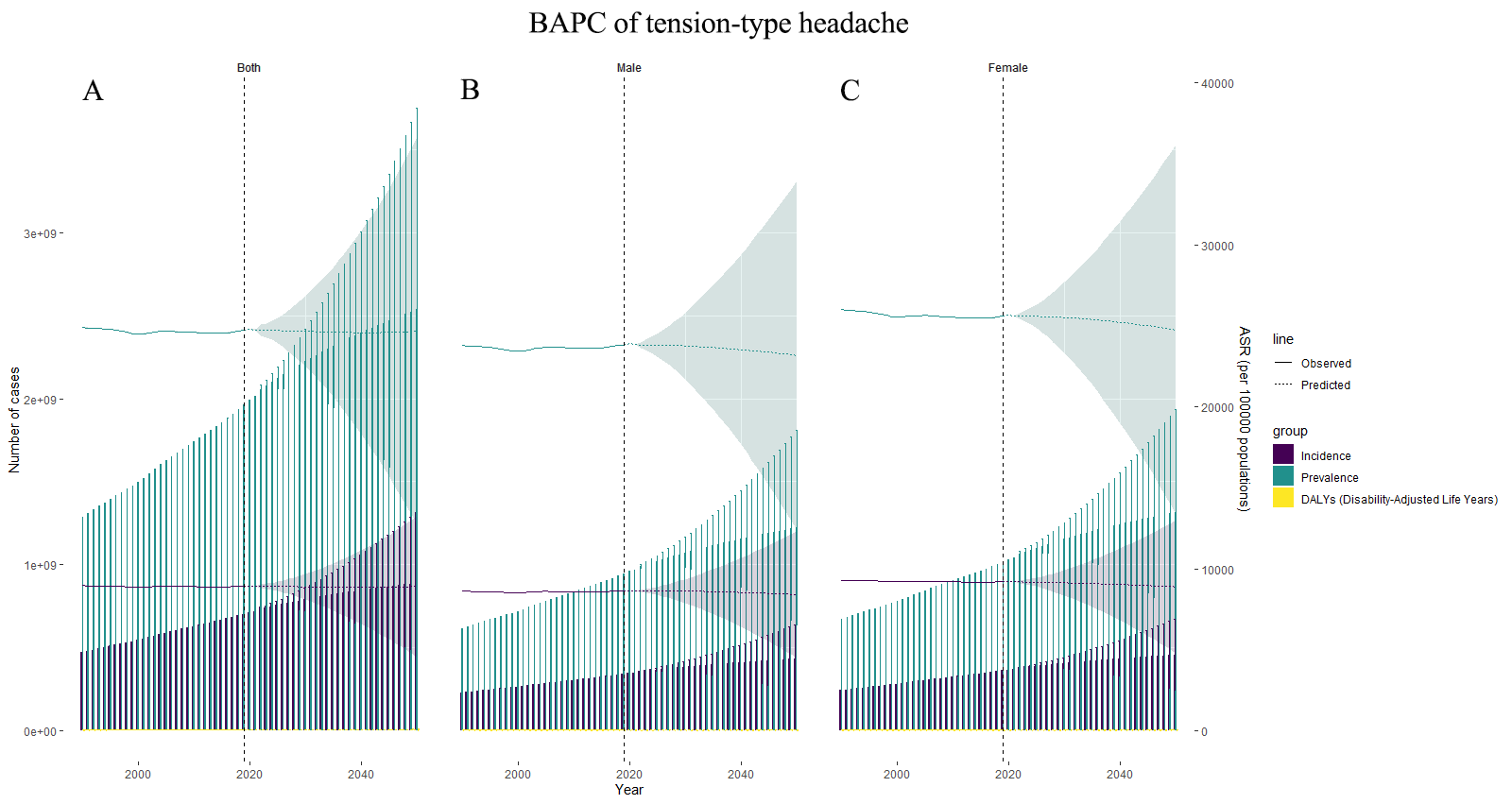

Supplement: Supplementary file 1 [file Data_Sheet_1.zip › Supporting Information/S17_Fig.tif]

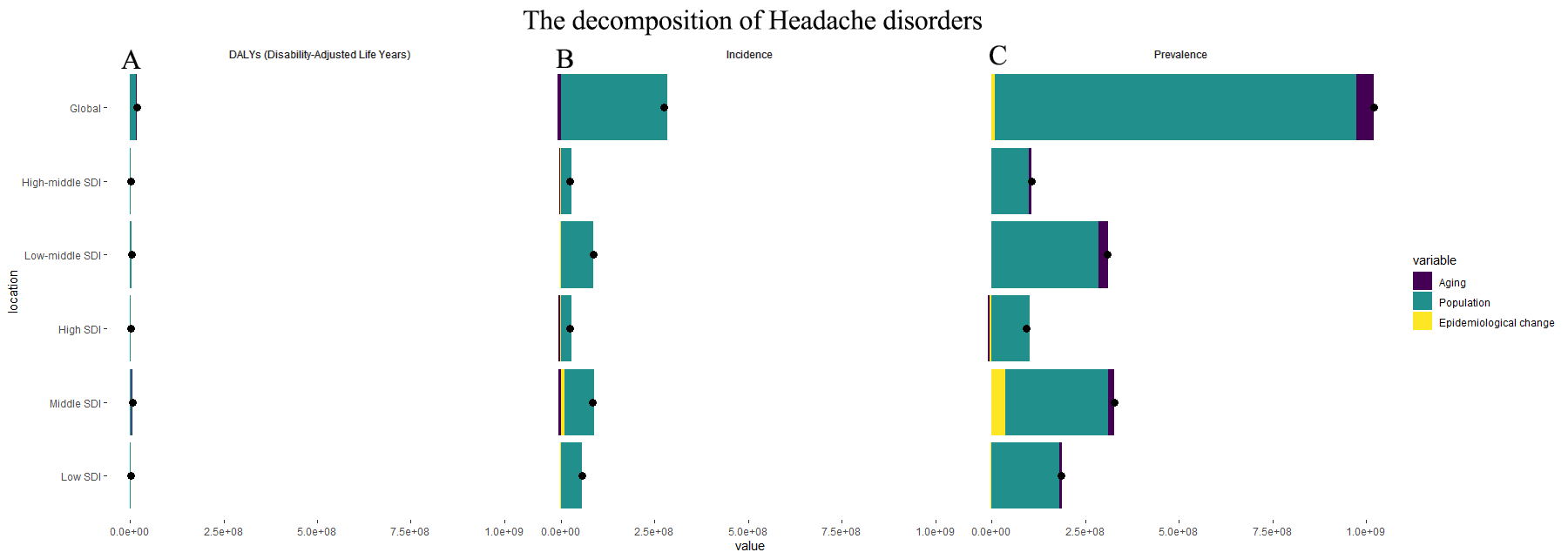

Supplement: Supplementary file 1 [file Data_Sheet_1.zip › Supporting Information/S18_Fig.tif]

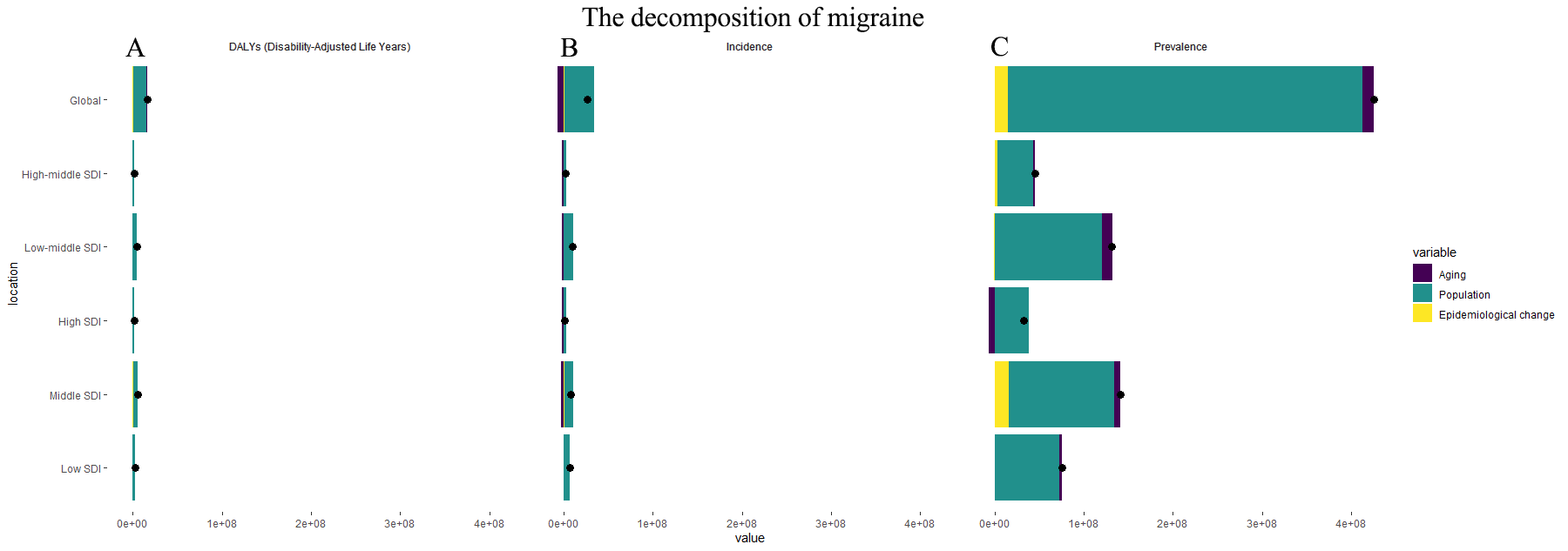

Supplement: Supplementary file 1 [file Data_Sheet_1.zip › Supporting Information/S19_Fig.tif]

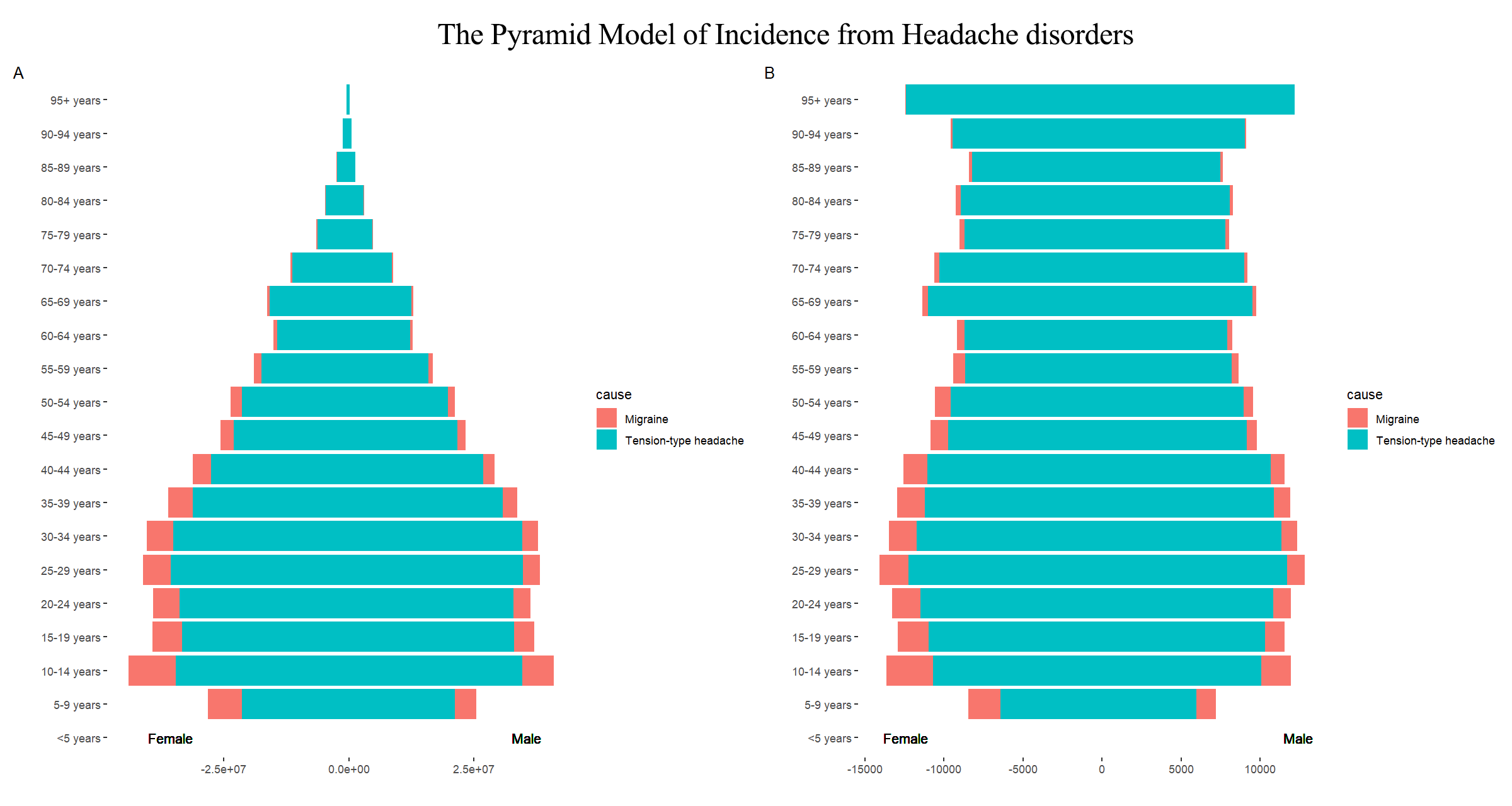

Supplement: Supplementary file 1 [file Data_Sheet_1.zip › Supporting Information/S1_Fig.tif]

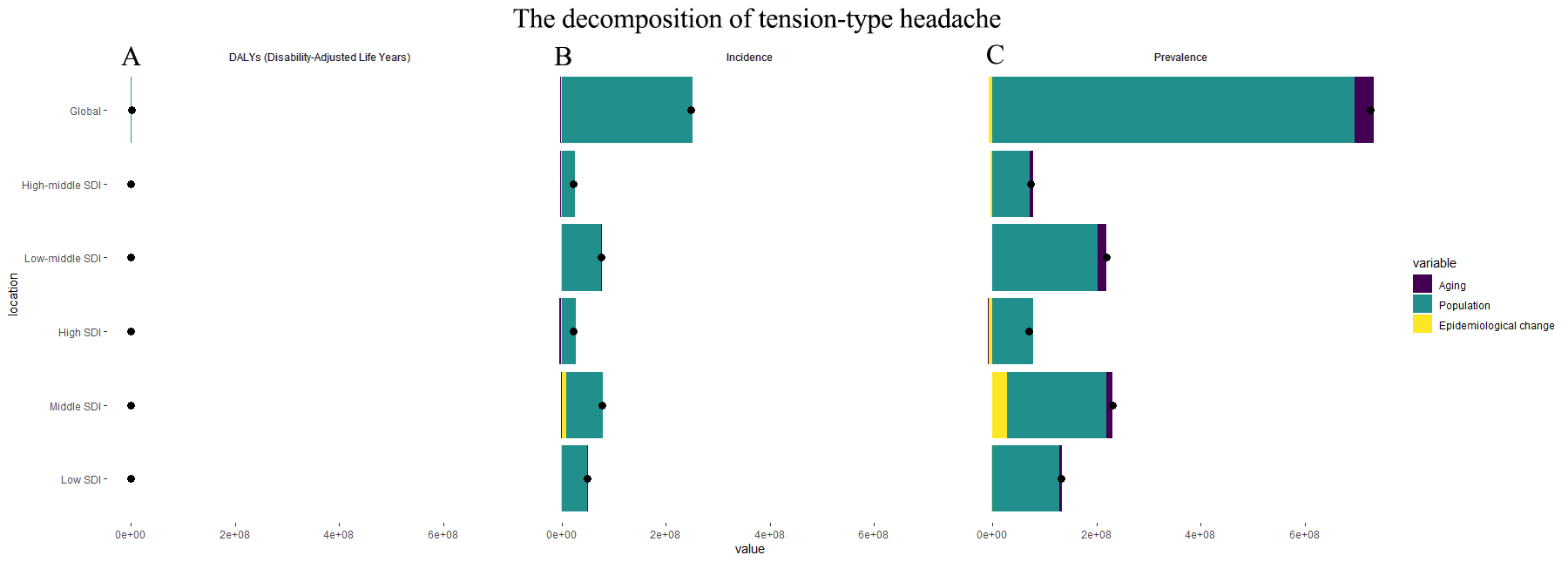

Supplement: Supplementary file 1 [file Data_Sheet_1.zip › Supporting Information/S20_Fig.tif]

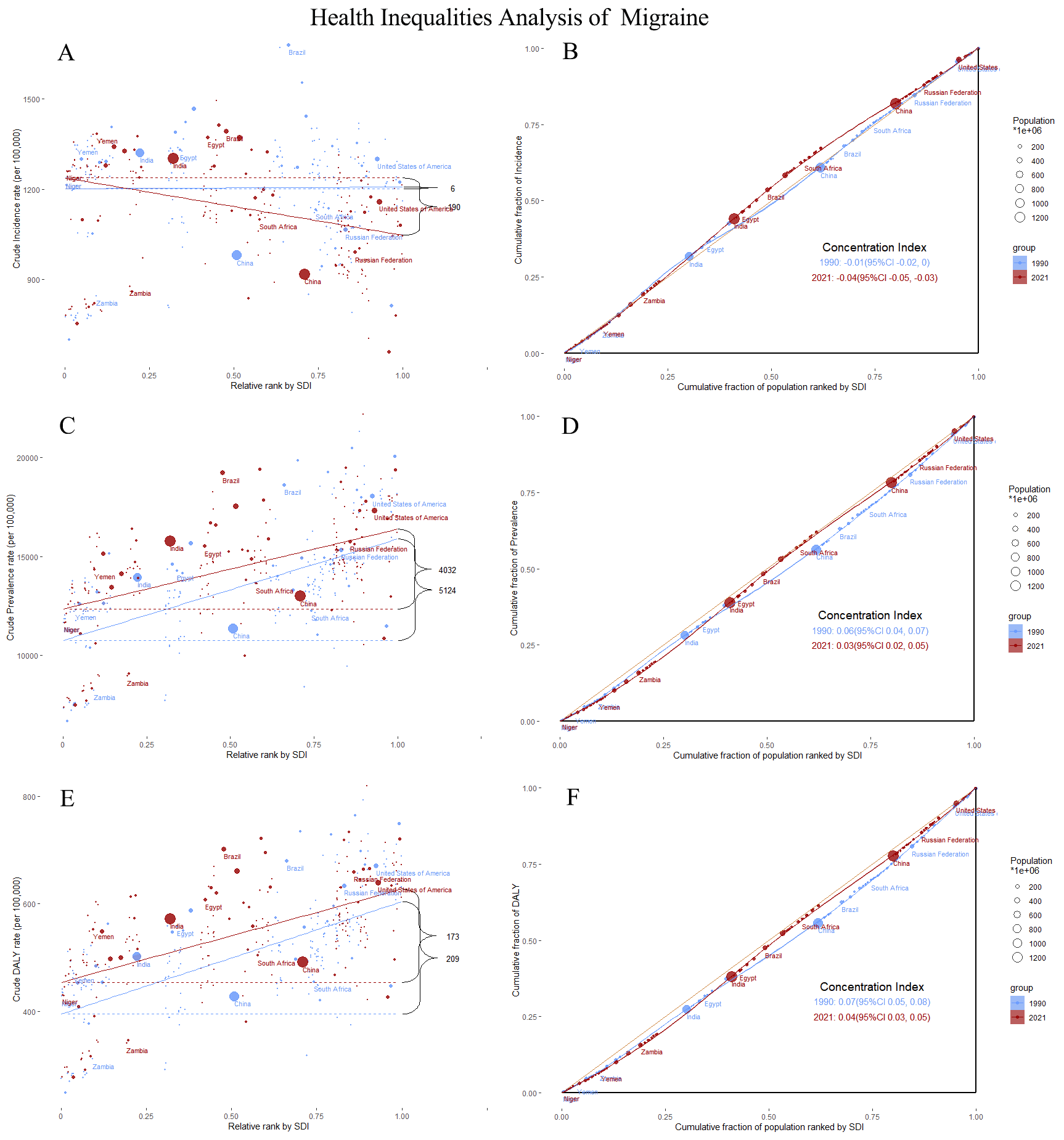

Supplement: Supplementary file 1 [file Data_Sheet_1.zip › Supporting Information/S21_Fig.tif]

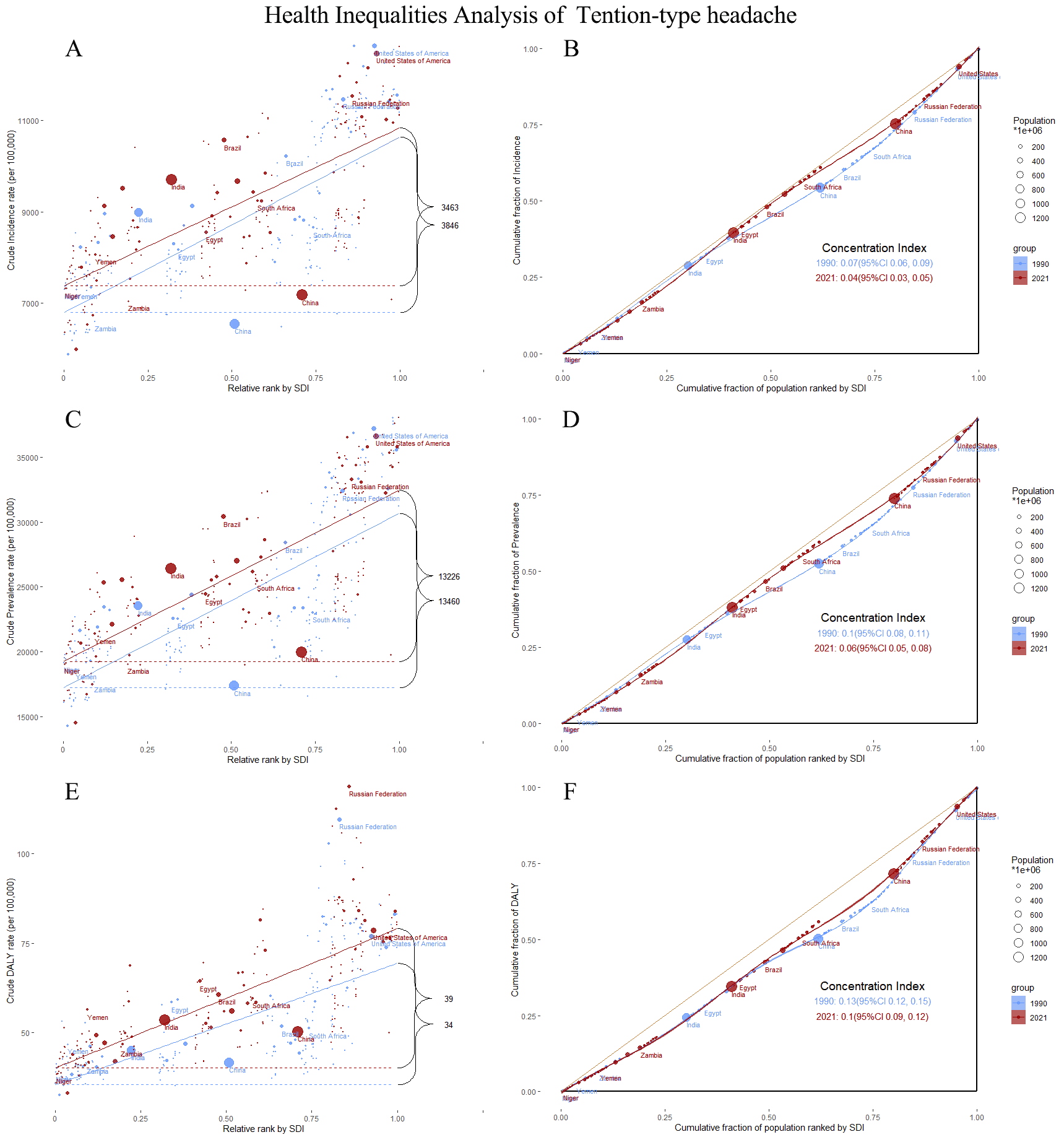

Supplement: Supplementary file 1 [file Data_Sheet_1.zip › Supporting Information/S22_Fig.tif]

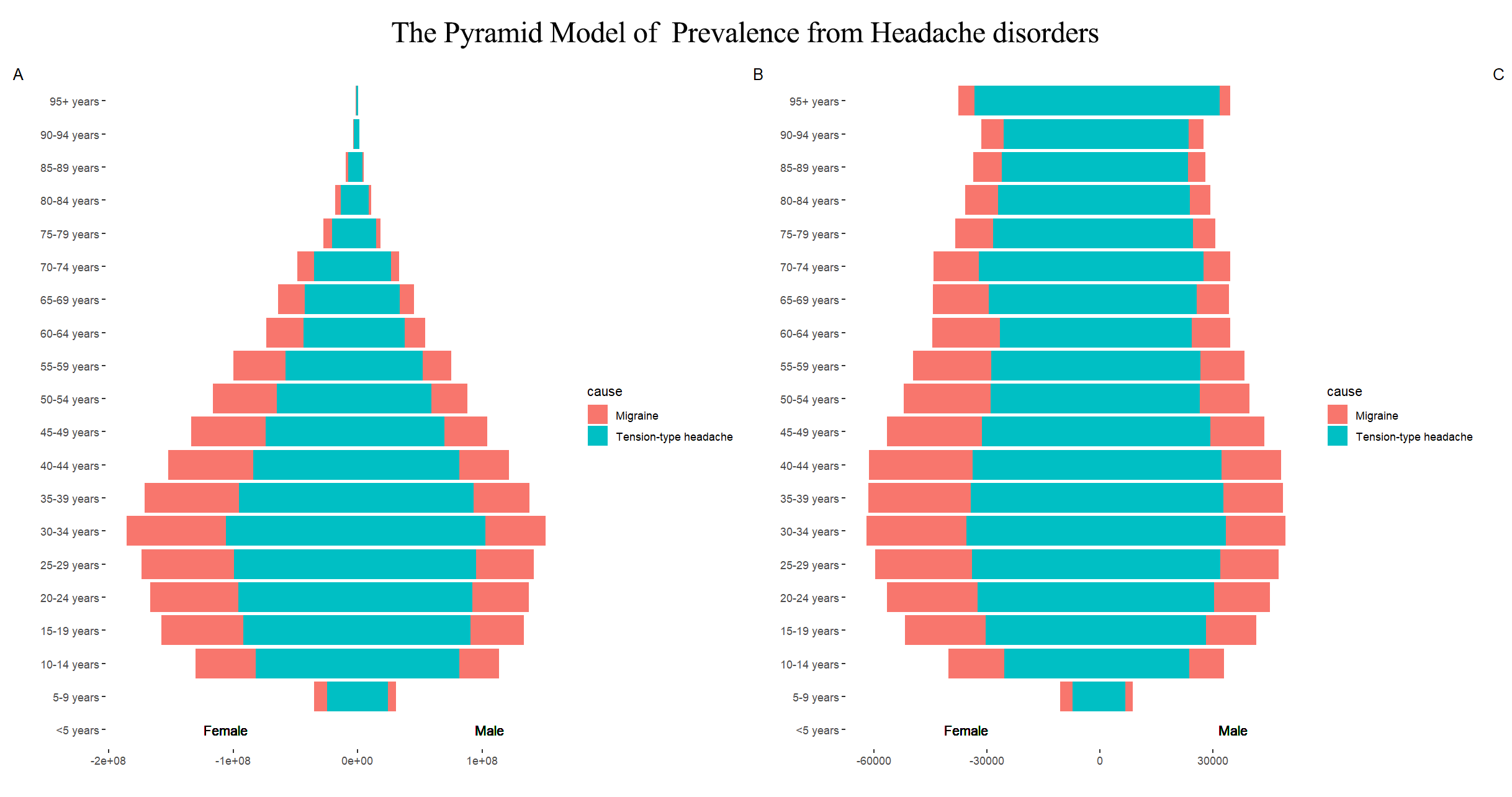

Supplement: Supplementary file 1 [file Data_Sheet_1.zip › Supporting Information/S2_Fig.tif]

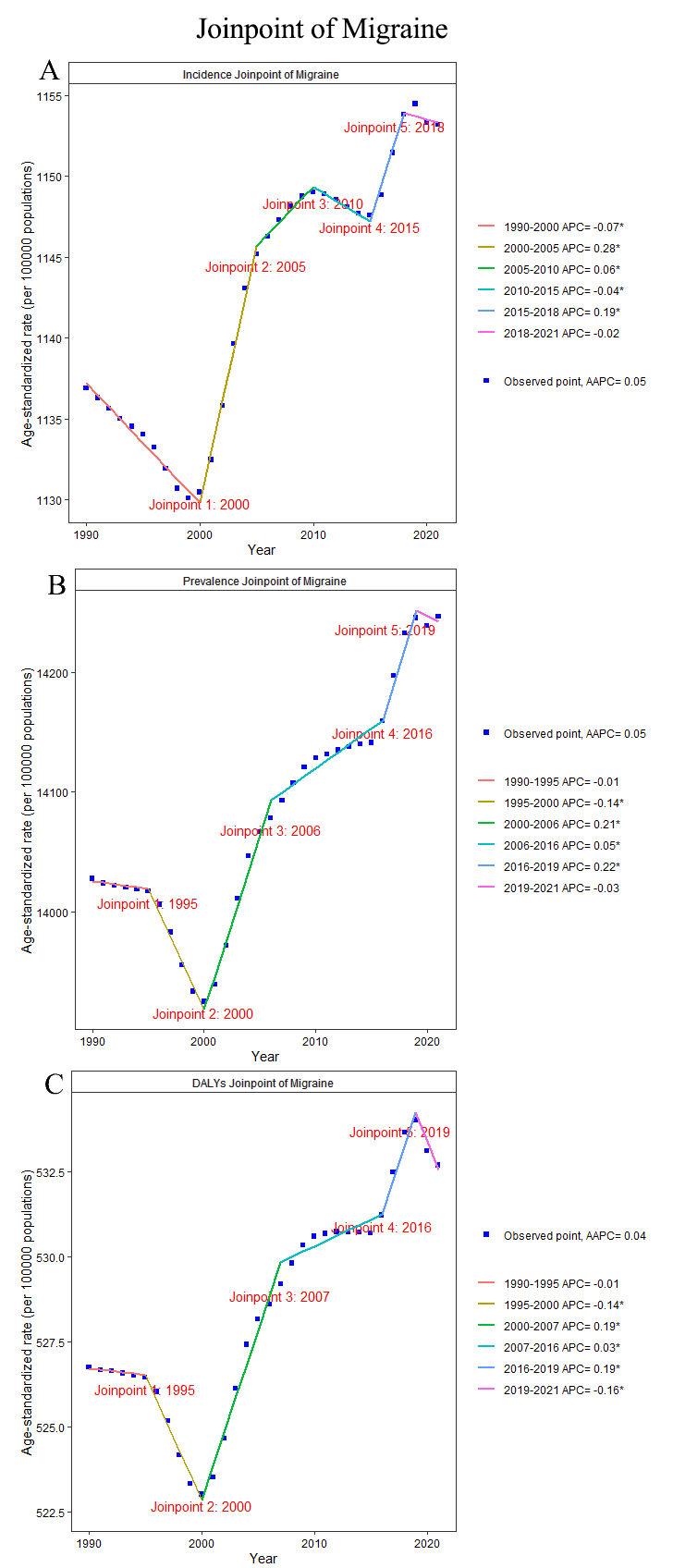

Supplement: Supplementary file 1 [file Data_Sheet_1.zip › Supporting Information/S3_Fig.tif]

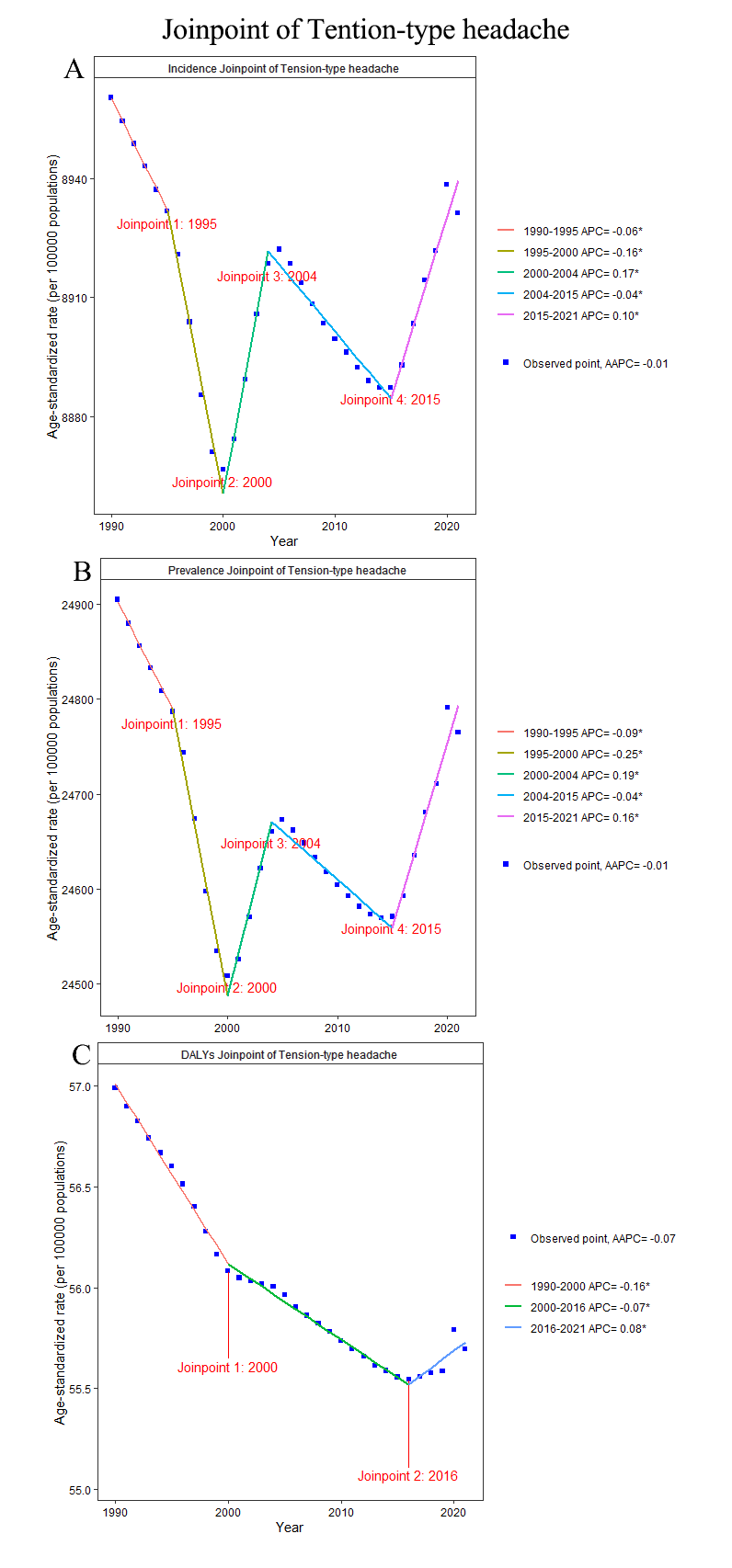

Supplement: Supplementary file 1 [file Data_Sheet_1.zip › Supporting Information/S4_Fig.tif]

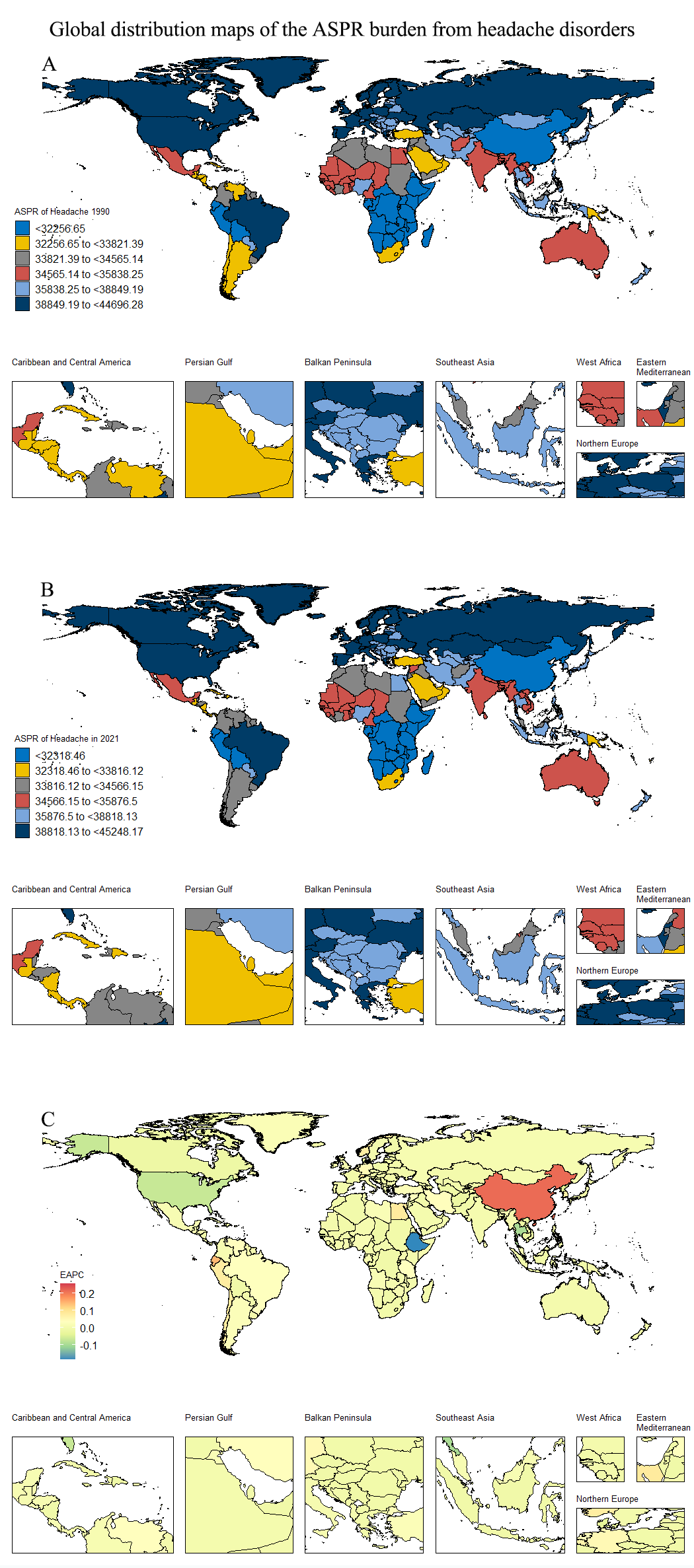

Supplement: Supplementary file 1 [file Data_Sheet_1.zip › Supporting Information/S5_Fig.tif]

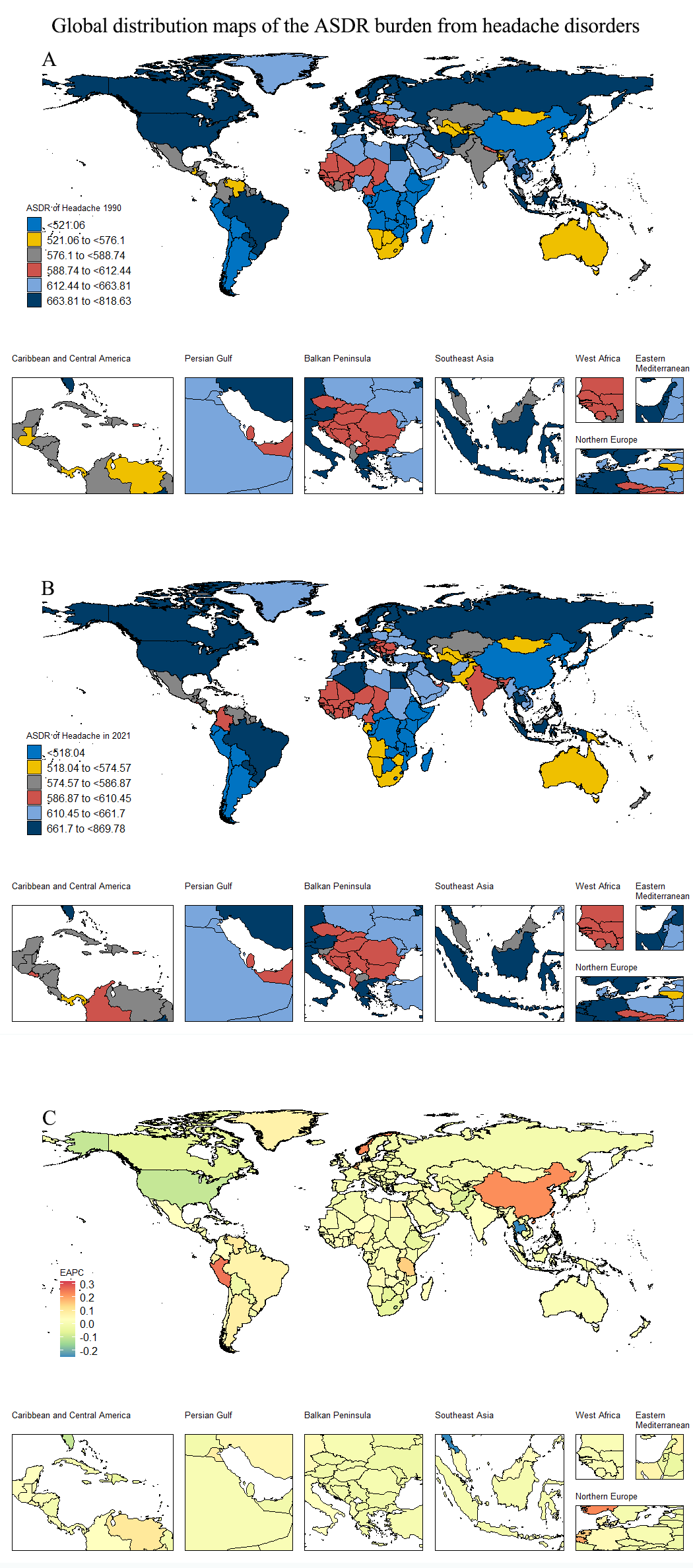

Supplement: Supplementary file 1 [file Data_Sheet_1.zip › Supporting Information/S6_Fig.tif]

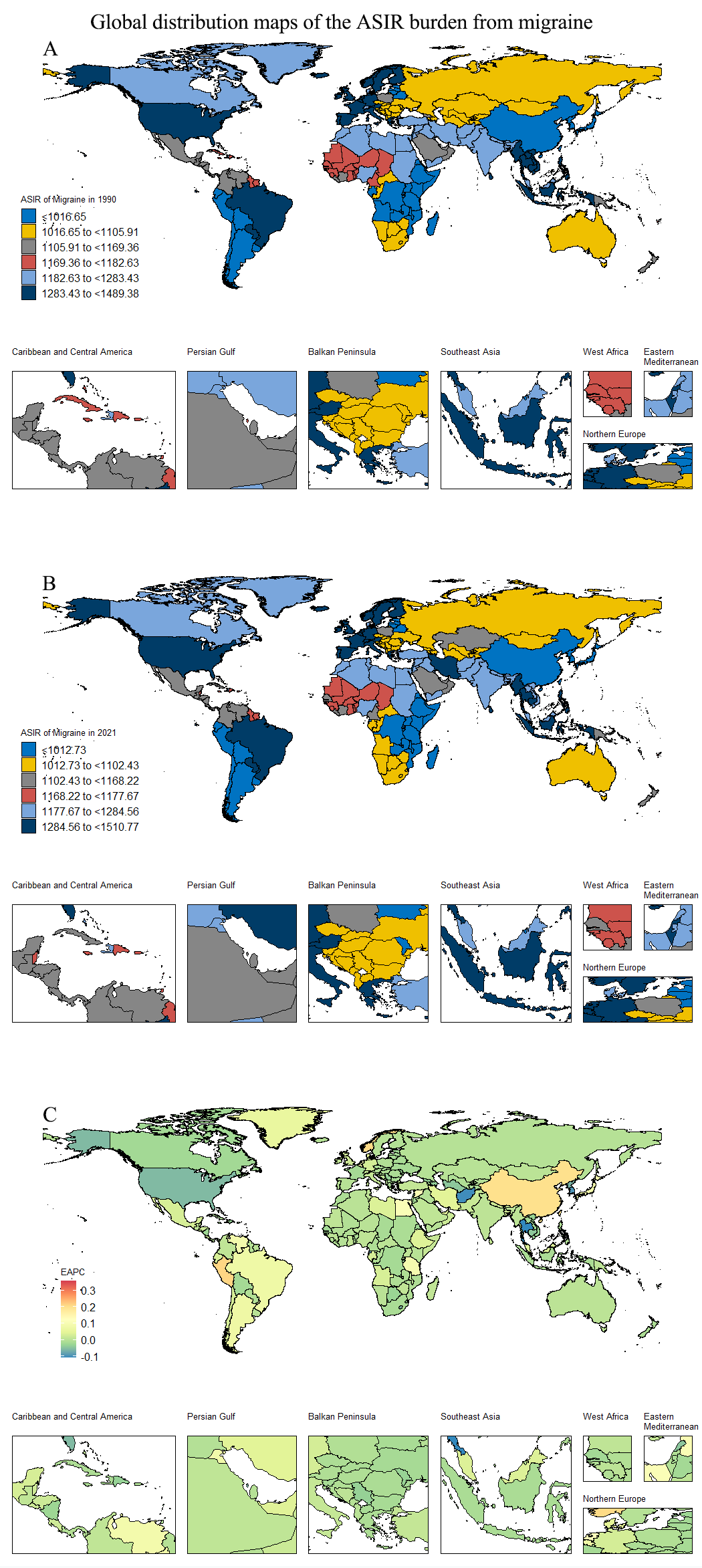

Supplement: Supplementary file 1 [file Data_Sheet_1.zip › Supporting Information/S7_Fig.tif]

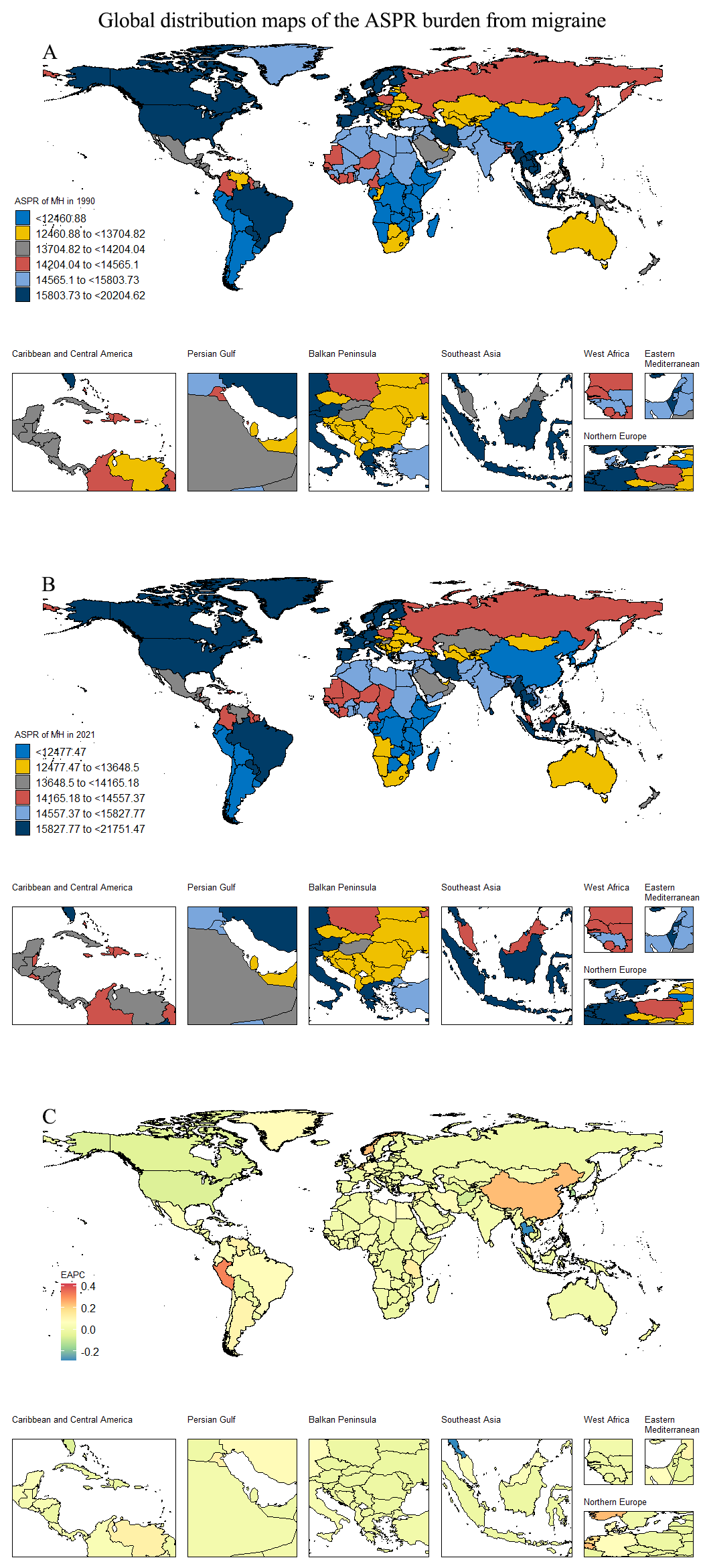

Supplement: Supplementary file 1 [file Data_Sheet_1.zip › Supporting Information/S8_Fig.tif]

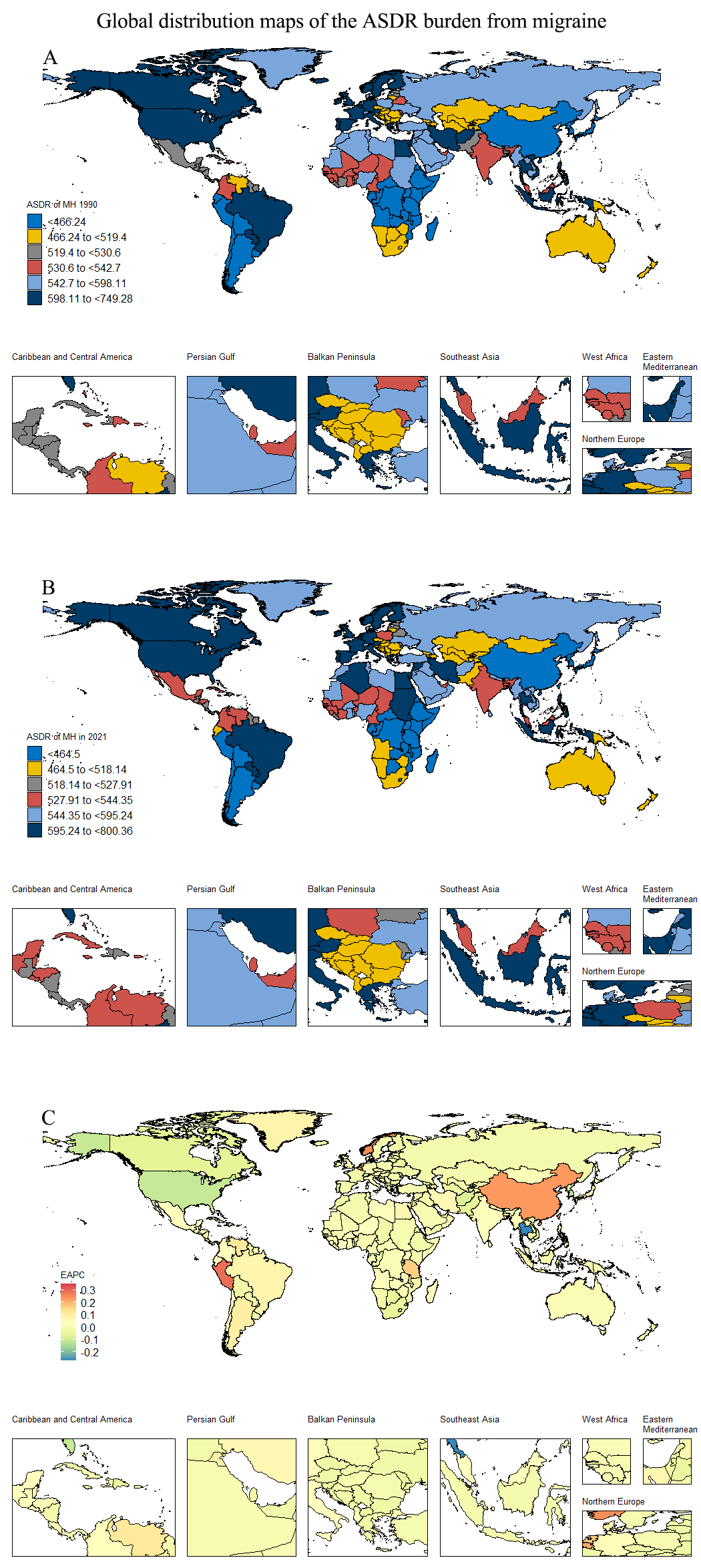

Supplement: Supplementary file 1 [file Data_Sheet_1.zip › Supporting Information/S9_Fig.tif]
